# Investigation of the network of preferred interactions in an artificial coiled-coil association using the peptide array technique

**DOI:** 10.3762/bjoc.8.71

**Published:** 2012-04-25

**Authors:** Raheleh Rezaei Araghi, Carsten C Mahrenholz, Rudolf Volkmer, Beate Koksch

**Affiliations:** 1 Institute of Chemistry and Biochemistry, Freie Universität Berlin, Takustrasse 3, 14195 Berlin, Germany; 2 Institute of Medical Immunology, Charité-Universitätsmedizin Berlin, Augustenburger Platz 1, 13353, Berlin, Germany

**Keywords:** β- and γ-amino acids, coiled coil, foldamer, screening libraries, SPOT technique

## Abstract

We screened a randomized library and identified natural peptides that bound selectively to a chimeric peptide containing α-, β- and γ-amino acids. The SPOT arrays provide a means for the systematic study of the possible interaction space accessible to the αβγ-chimera. The mutational analysis reveals the dependence of the binding affinities of α-peptides to the αβγ-chimera, on the hydrophobicity and bulkiness of the side chains at the corresponding hydrophobic interface. The stability of the resulting heteroassemblies was further confirmed in solution by CD and thermal denaturation.

## Introduction

Coiled-coil domains, which consist of two or more α-helices, are the most common representatives of α-helix-mediated protein–protein interactions, which regulate many important biological pathways [1]. Coiled coils have several advantageous features that, on the one hand, allow them to fulfill a wide range of important cellular functions [2] and, on the other hand, make them ideal building blocks in protein design: They are ubiquitous proteins that have the ability to oligomerize with high selectivity, forming stable multimers with strong interhelical interactions. Recently, their potential as drug targets has become the focus of medical research [3]. Their effectiveness in the successful inhibition of membrane fusion proteins of viruses, such as HIV [4] and avian influenza [5], also supports the concept of rational drug design based on coiled-coil proteins [6]. In this context, the use of unnatural amino acids in peptidomimetics is advisable, to enhance enzymatic stability, limit conformational flexibility, and improve pharmacodynamics and bioavailability [7]. In order to manipulate helix-mediated interactions to achieve high specificity levels, a pioneering approach is to design helical foldameric sequences containing β- and γ-amino acids. Foldamers are shown to form, successfully, a variety of conformations at secondary, tertiary, and quaternary structure levels [8–10]. In spite of the increasing number of helical assemblies made of peptidic foldamers, the combination of artificial oligomers with natural peptides remains a challenge. The main difficulties arise in the prediction of a suitable side-chain composition and the geometry of the foldameric binding groove that interacts with α-peptides [11]. Therefore, elucidating the side-chain compositions responsible for selective intermolecular interactions in an otherwise natural coiled-coil assembly should facilitate the design of helix–helix interaction motifs. We broadly surveyed interaction properties in order to improve the association between artificial and natural patterns by means of SPOT technology, which is a simple high-throughput method shown to be useful for the characterization of intermolecular domains in general and coiled coils in particular at the amino-acid level [12–15]. This method assisted in the mapping of α-residues of a natural peptide strand that interact with key β- and γ-amino acids of a chimeric peptide. A wide range of analogues of wild-type α-partners were synthesized and analyzed in order to evaluate affinity, selectivity, and the binding determinants of the αβγ-chimeric backbone. Further, the stability and the stoichiometry of selected sequences were examined by CD and SEC in solution.

## Results and Discussion

**Figure 1:**
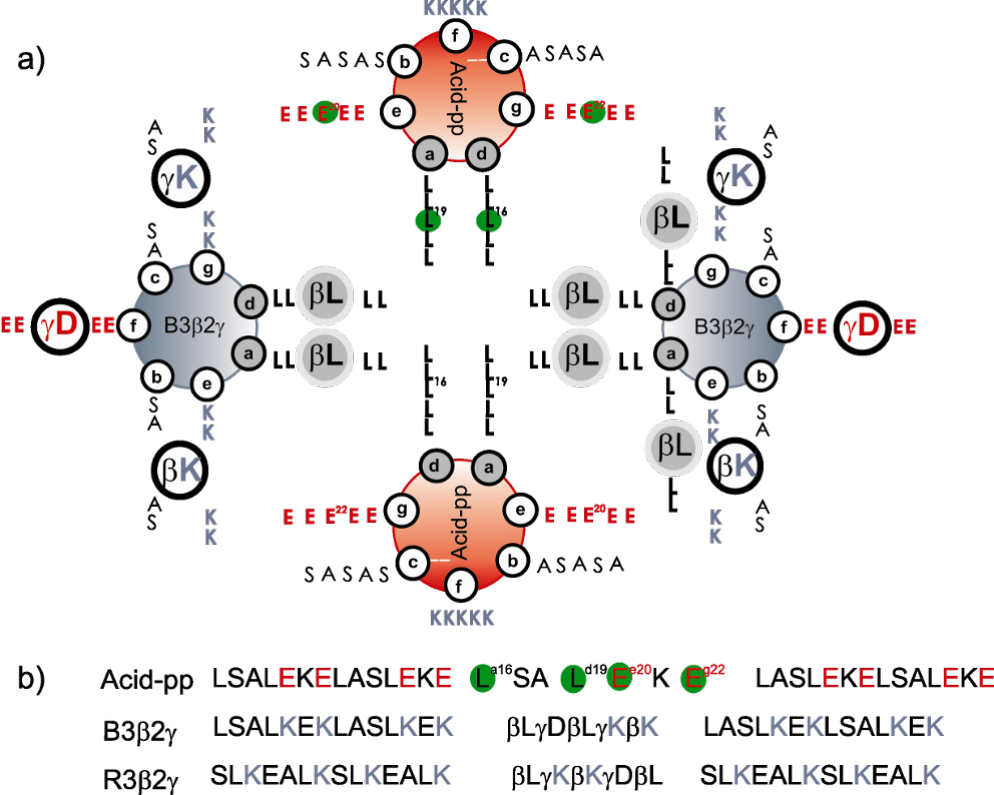
(a) Helical wheel representation of the tetrameric Acid-pp/B3β2γ helix bundle, (b) sequences of Acid-pp, B3β2γ, R3β2γ. Red and blue letters indicate the acidic residues in Acid-pp and the basic residues in B3β2γ and R3β2γ, respectively. The randomized positions are designated by green circles.

**Screening system:** Coiled coils are a highly populated class of protein-folding motifs that exhibit a distinctive heptad repeat sequence, conventionally labeled with the letters *a*–*g* [16–17]. The set of hydrophobic residues at the first (i.e., *a*) and fourth (i.e., *d*) positions pack into the coiled-coil interfaces and play the main role in helical association, while the *e* and *g* positions frequently consist of polar or charged residues forming the electrostatic interface. A preliminary structural investigation revealed that one such characteristic heptad can be substituted by a pentad repeat sequence of alternating β- and γ-amino acids, while retaining folding stability [18–19]. The backbone-engineered coiled-coil system comprises two peptides: A glutamic acid-rich α-poly peptide (Acid-pp) and a lysine-rich chimeric B3β2γ sequence. These systems have a high propensity for heterooligomerization (Figure 1). In B3β2γ, the two central turns of the α-helix are substituted by a pentad of alternating β- and γ-amino acids. Our previous study revealed the heteromeric assembly of natural and chimeric sequences with 1:1 stoichiometry [18]. The heterooligomerization is driven by the burial of the hydrophobic surface area and is directed by electrostatic interactions between charged residues that flank the hydrophobic core. However, substituting an α-heptad with a pentad of β- and γ-amino acids has structural consequences, such as disruption of the local packing, or conformational chaos due to the loss of one peptide bond and therewith one H-bond donor and one H-bond acceptor. More recently, it has been shown that a careful choice of side chains can provide key residue contacts and a sufficient number of van der Waals interactions to enable the chimeric sequence to form helical interfaces interacting with a native peptidic partner [20]. These instructive investigations have revealed that side-chain and backbone characteristics are tuneable elements for control of the interaction between αβγ-chimera and their native partners. To extend these studies, in the current report, we screen the “interaction space” of chimeric coiled coils using a large library of native peptides in order to search for more efficient interacting partners.

**Figure 2:**
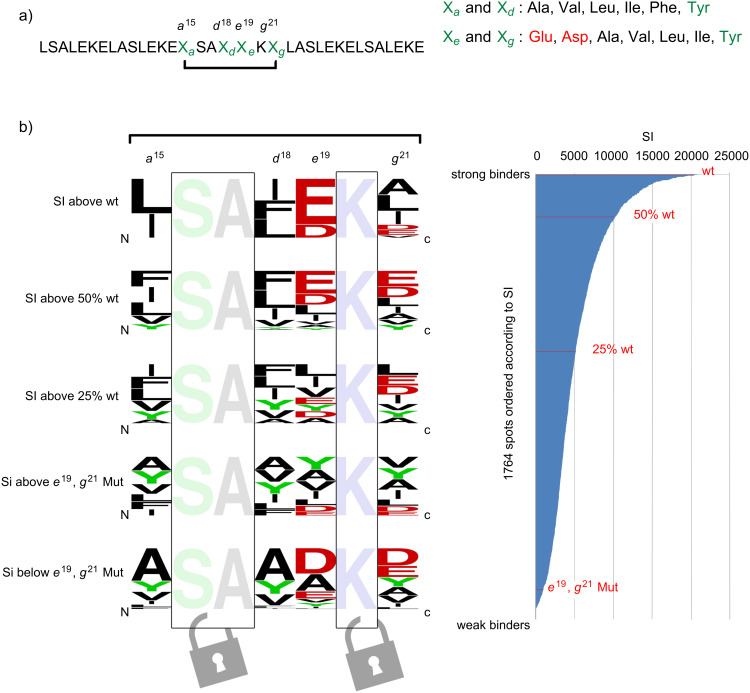
(a) Sequence for random mutation resulting in 1764 spots. The randomized positions are denoted by X*_a_*, X*_d_*, X*_e_*, and X*_g_*, and the amino acids selected for randomization are listed on the right. (b) The sequence logos (left) indicate the frequencies of specific *a*/*d*/*e*/*g* substitutions within a class (the larger the letter, the higher the frequency). The mutated positions *a*, *d*, *e*, and *g* are denoted by 15, 18, 19, and 21, respectively. The graph (right) displays the signal intensity (SI) of the 1764 spots and is ordered from strong to weak binders. Indicated are the four interaction classes grouped according to SIs relative to the Acid-pp wt SI and the *e*^19^,*g*^21^ mutant.

**Library design and synthesis:** To investigate the Acid-pp–chimera interaction, a peptide array (1764 spots), featuring multiple substitutions at positions *a*/*d*/*e*/*g* of the central heptad of the *wt* Acid-pp sequence (Figure 2a), was created on cellulose membrane and probed for binding to the αβγ-chimera. The chimeric sequence B3β2γ, containing three β- and two γ-amino acids, was synthesized by standard solid-phase peptide synthesis and labeled at the N-terminus with the fluorophore 5(6)-tetramethylrhodamine (TAMRA). As described above, the chimera has a modified pentad (β- and γ-amino acids) at the center of its 31-residue sequence (positions 15–19). Thus, the positions in the complementary heptad of Acid-pp (positions 15–21) were mutated, as shown in Figure 1a and Figure 1b. In order to investigate the contact elements on the natural peptide that are crucial for the coiled-coil interaction with the chimeric sequence, the residues located close to and within the interhelical core (*a*,*d*,*e*,*g*), which are most important for molecular recognition, were mutated. More specifically, hydrophobic (*a*^15^,*d*^18^) and electrostatic (*e*^19^,*g*^21^) positions were mutated simultaneously (Figure 2). The 35mer mutants on the membrane only differ in the positions that interact with side chains of the β- and γ-residues.

Mutational analysis of the selected positions (*a*^15^,*d*^18^,*e*^19^ and *g*^21^) was carried out with a chosen set of amino acids (Figure 2a, right) to characterize the suitable side-chain composition for optimal interaction with the βγ-foldameric pattern on the chimeric αβγ-sequence.

Thereby, the most favorable amino acid side chains at each key position were identified. Since the *a* and *d* positions are typically occupied by hydrophobic residues in most of the naturally occurring coiled-coil sequences, the mutations at *a*^15^ and *d*^18^ positions incorporated only hydrophobic amino acids, including the sterically bulky ones. To support heteroassembly between the αβγ-chimeric sequences and their natural counterparts, the complementary negative side chains of Glu and Asp residues as well as a series of hydrophobic side chains were considered at the *e*^19^ and *g*^21^ positions. Interactions between the chimera and immobilized α-mutants were measured by using a peptide array assay.

**Figure 3:**
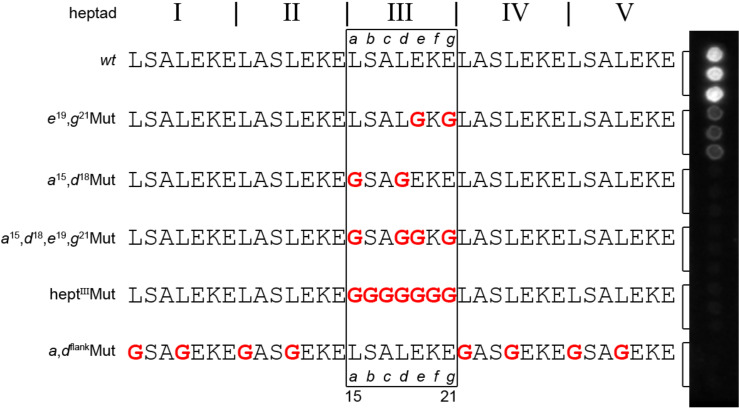
Glycine scanning of Acid-pp sequences. The substituted glycines are highlighted in red. Each sequence is followed by its corresponding spot (three replicas each). White spots indicate interactions between cellulose membrane-bound variants of Acid-pp and the TAMRA-labeled chimera.

Additionally, to ensure that the chimera interacts with the surface-bound peptides at the aforementioned hydrophobic (*a* and *d*) and electrostatic (*e* and *g*) positions, several positions relevant to coiled-coil binding were replaced with glycine. As depicted in Figure 3, both/either (i) hydrophobic (*a*^15^,*d*^18^) and/or electrostatic (*e*^19^,*g*^21^) positions of the central heptad or (ii) essential hydrophobic positions in the flanking heptads were substituted with glycine. This set of sequences was used to evaluate the quality of interaction between the side chains of immobilized α-mutants and the complementary αβγ-chimeric sequence.

**Spot analysis:** A peptide array containing the multiple substitutions was incubated with the chimera, and spot signal intensities of the resulting heteromeric associations were measured and evaluated. The measured signal intensities (SI) obtained from the TAMRA-labeled modified sequence interacting selectively with immobilized α-peptides, were classified as described above. Remarkably, despite their limited sequence variability, the α-mutants exhibited various degrees of binding affinity to the chimera. Based on the SI values, the α-mutants were classified into five interaction groups. As depicted in Figure 2 on the right-hand side, sequences with equal or slightly higher SI values compared to Acid-pp were classified as strong binders (only 22 representatives). The second and third classes contain the mutants with SI values lower than those of the strong binders but still above 50% and 25% of that of Acid-pp, respectively. The poor binders have SI values below 25% of that of Acid-pp. This group also contains the aforementioned glycine-mutants. For each class we computed the residue frequencies at the mutated positions *a*, *d*, *e*, and *g* of the central heptad and summarized the results as sequence logos (Figure 2, left). At each position, the residues are arranged in order of predominance from top to bottom, and the mutants we selected are named after the respective combinations of four mutated residues. The frequencies of specific side chains in the recognition domain indicate the preferred interactions between the β- and γ-amino acids and the complementary side chains of the natural α-partners. These results suggest that only a few mutants are able to interact efficiently with B3β2γ.

The glycine-scan (Figure 3) reveals the nature of the chimeric coiled-coil interaction. Only the acid-pp *wt* shows strong binding to B3β2γ. If the hydrophobic or electrostatic regions essential for coiled-coil binding are blocked by glycine, the signal breaks down. As expected, the glycine scanning of the hydrophobic residues at *a* and *d* positions of the flanking heptads (*a*,*d*^flank^Mut) results in a diminishing of interactions between chimera and the natural partner peptide. Remarkably, the replacement of only two hydrophobic side chains located at the central heptad is equally destabilizing for the entire assembly, indicating the key role of the interhelical interactions between the third heptad of the native sequences and foldameric section of the chimera. Manipulation of the interacting native amino acids on the third heptad (*a*^15^,*d*^18^Mut), (*e*^19^,*g*^21^Mut), (*a*^15^,*d*^18^,*e*^19^,*g*^21^Mut) resulted in a loss of binding, which strongly suggests that the quaternary structure does not only tolerate but is in fact dependent on the interaction of the central heptad of natural peptides with side chains of the unnatural amino acids on the chimera.

As a negative control, the binding affinity of the modified sequences was tested against a randomly designed αβγ-chimeric sequence, R3β2γ (Figure 1b). In the R3β2γ sequence, all of the amino acids including β- and γ-residues are randomly distributed. The screening of the peptide library against this chimeric peptide results in a poor binding profile. Interestingly, and in contrast to the randomly designed R3β2γ, the sequences on the membrane show remarkable selectivity in the binding to B3β2γ.

**Figure 4:**
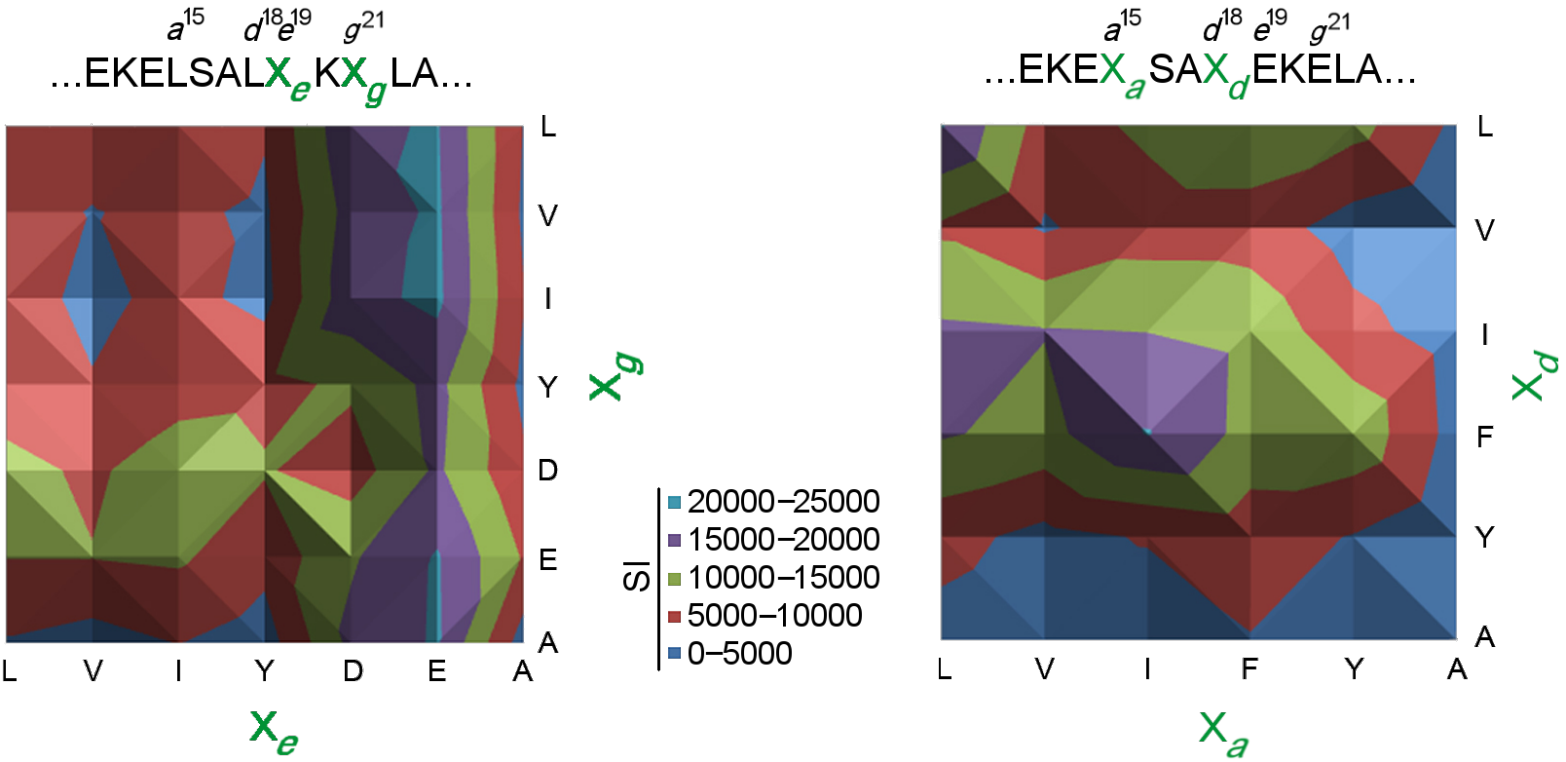
Heat-map diagrams depicting the quantitatively measured SIs for Acid-pp sequences containing mutations at positions *e*^19^ and *g*^21^ (left) and at positions *a*^15^ and *d*^18^ (right) in heptad III. The SIs corresponding to each color are displayed between the heat maps.

In general, a direct relation exists between the observed light intensity and the heteroassociation between chimera and mutants on the membrane. More importantly, the implicit binding affinities are highly consistent with previously published data [18]. In our previous study we reported a gradual destabilization of the chimeric coiled-coil assembly with gradual truncation of the β- and γ-side chains at the hydrophobic core due to a loss of hydrophobic interactions between the αβγ-peptide and its natural α-partner. Similarly, the interactions between the peptides on the array and the chimera decrease drastically in sequences presenting the shorter side chains of Val and Ala at *a* and *d* positions, compared to mutants with the longer and more bulky side chains of Leu, Ile, and Phe (Figure 4, right). Furthermore, the negligible binding affinities between the chimera and the control sequences, in which the hydrophobic and electrostatic residues were replaced with glycine residues, indicate that B3β2γ prefers mutants that provide sufficient side-chain–side-chain contact.

The peptide array detected many new side-chain combinations, the investigation of which provides useful insights into the complex contact networks of an αβγ-chimeric folding motif. Enlarging the interior cavity of coiled coils by positioning the backbone extended β- and γ-amino acids in the hydrophobic core necessitates compatible coverage. This requirement is confirmed by the sequence logo for strong binders (Figure 2b, first on top): A stronger peptide–peptide interaction was observed between the chimeric pattern of B3β2γ and the α-mutants with large and bulky hydrophobic residues, for instance the aromatic Phe, the long and bulky Leu, and the β-branched Ile.

Remarkably, the preference for hydrophobic residues is highly position-dependent (Figure 3b and Figure 4); Phe is favored at the *d* position, while Leu is more prevalent in the *a* position. Moreover, substitution of a single hydrophobic position with tyrosine (L^15^Y) in the Y*^a^*L*^d^*E*^e^*E*^g^* mutant results in a significant reduction in the spot intensity compared to that of *wt* Acid-pp. As already discussed, despite the similarity in size between Phe and Tyr residues, they exhibit prominent differences in the coiled-coil formation that are probably due to the destabilizing orientation of the polar hydroxyl group towards the hydrophobic core [21]. The clear decrease in binding affinity between chimera and Tyr-comprising mutants shows that the side chains at the artificial interface of the helix bundle experience an environment similar to that of natural coiled coils.

Another important observation is that, although they provide sufficient hydrophobicity at the interhelical domain, the mutants with two Phe residues are among the weaker binders (Figure 4, right). This is also true for the β-branched Ile; substitution with two Ile residues results in a medium binding affinity (Figure 4). This can be explained by the fact that, similarly to that of native coiled coils, the chimeric hydrophobic core is disrupted by excessively bulky side chains [22]. However, the preference for specific hydrophobic side chains in *a* and *d* positions and of unique combinations thereof indicates that the selection of residues in the hydrophobic core is determined not only by side-chain hydrophobicity but also by side-chain packing. The packing geometry is thus an important aspect influencing the stability of artificial coiled coils. These results additionally confirm the impact of electrostatic interactions at the *e* and *g* positions. The SI values of sequences mutated in these positions show that shortening of the negatively charged side chain in the case of the Glu*^e^*^19^Asp exchange, results in a general decline in binding affinity for almost all mutants presented on the membrane (Figure 2, left and Figure 4, left). However, there is a discrepancy between the two core-flanking positions; the *e* position was found to be significantly more sensitive to replacement than the supposedly similar *g* position. This fact has also been observed and reported for natural coiled coils [23]. The different interaction profiles of the side chains at *e* and *g* positions (Figure 4, left) can be caused by the asymmetrical geometry of complementary Lys side chains on the βγ-foldameric interaction partner. A further interesting observation is that hydrophobic residues populate the core-flanking positions, which suggests that the side chains at these positions lead to an extension of the hydrophobic core.

**Figure 5:**
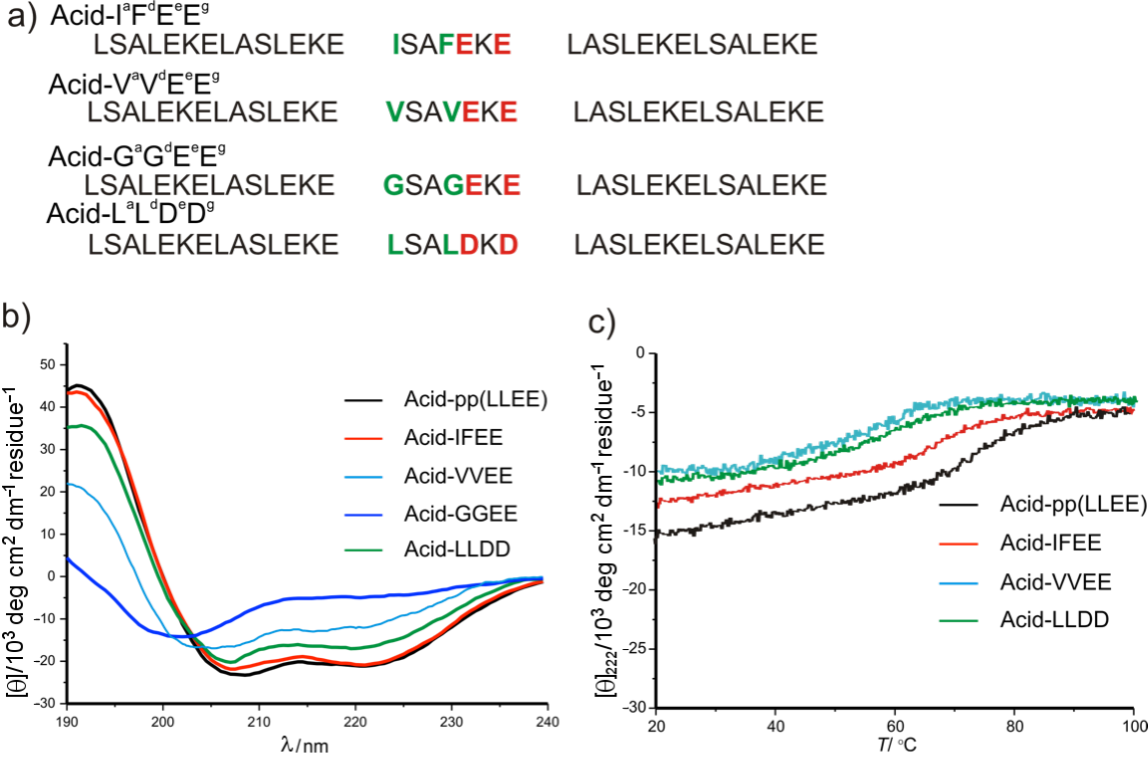
(a) The complete sequences of the selected α-mutants. (b) CD and (c) thermal denaturation spectra of an equimolar mixture of B3β2γ and the α-mutants (25 μM of peptide concentration in phosphate buffer 50mM in presence of 250 mM GndHCl).

**Solution study:** To gain more insight into the relationship between hetero-selective binding and structural stability, several mutants from different classes were studied further in solution (Figure 5a). These sequences were synthesized on resin, purified, and finally investigated by CD and size-exclusion chromatography. We chose I*^a^*F*^d^*E*^e^*E*^g^* as a representative of the strong binders to be tested in solution because of the frequently repeated positioning of Phe in the hydrophobic core, that is, more specifically at the *d* positions. Moreover, the behavior of V*^a^*V*^d^*E*^e^*E*^g^* and G*^a^*G*^d^*E*^e^*E*^g^*, belonging to the medium and poor binder classes, respectively, was tested in solution (Figure 4). Another mutant we selected is L*^a^*L*^d^*D*^e^*D*^g^*, in which both *e*^19^ and *g*^21^ positions are occupied by Asp, which, according to the observed SIs, leads to weaker binding affinity to the chimera compared to Acid-pp.

An extremely weak interaction between B3β2γ and G*^a^*G*^d^*E*^e^*E*^g^*, indicated by a low spot signal intensity, was further confirmed in solution by a drastic decrease in helical content and structural stability (Figure 5b). This result identifies the side chains at *a*^16^ and *d*^19^ positions and their complementary β^3^Leu side chains on the chimera as hot spots in the chimeric recognition motif; interaction with these residues could lead to an increase in binding selectivity and stability.

An equimolar solution of B3β2γ/L*^a^*L*^d^*D*^e^*D*^g^* and B3β2γ/V*^a^*V*^d^*E*^e^*E*^g^* showed medium spot intensities (SIs of 7568 and 4487, respectively) with minima at 222 nm and 208 nm, which are less intense when compared with B3β2γ/Acid-pp (Figure 5a). Furthermore, the thermal melt *T*_m_ values of B3β2γ/L*^a^*L*^d^*D*^e^*D*^g^* (*T*_m_ = 64 °C) and B3β2γ/V*^a^*V*^d^*E*^e^*E*^g^* (*T*_m_ = 52 °C) also dropped in comparison to the parental system (Figure 5c). As expected, shortening of the side chains in the hydrophobic core, by replacing Leu side chains with Val in V*^a^*V*^d^*E*^e^*E*^g^*, had a more pronounced destabilizing effect on selectivity and stability of the resulting quaternary structure than did substitution of Glu side chains with Asp in core-flanking positions in the L*^a^*L*^d^*D*^e^*D*^g^* mutant. This fact is also reflected in their different spot intensities; the signal intensity of the L*^a^*L*^d^*D*^e^*D*^g^* mutant is almost half of that observed for V*^a^*V*^d^*E*^e^*E*^g^*.

In an analogous manner, the highly intense spot provided by interaction of the I*^a^*F*^d^*E*^e^*E*^g^* mutant with B3β2γ was confirmed further in solution by the intensive canonical minima of the α-helical coiled-coil structure at 222 and 208 nm and the relatively high thermal stability (Figure 5b, Figure 5c). The combination of Phe at *d* and Ile at *a* positions resulted in a significantly high binding affinity between I*^a^*F*^d^*E*^e^*E*^g^* and the chimera. Thermal denaturation of an equimolar mixture of B3β2γ and I*^a^*F*^d^*E*^e^*E*^g^* resulted in relatively high *T*_m_ values of 70 °C, which are close to those of an equimolar mixture of B3β2γ and Acid-pp (*T*_m_ values of 74 °C).

In order to compare both the packing effects and the burial of the hydrophobic surface of various side-chain compositions, the oligomerization states of the corresponding helix bundles of B3β2γ with native mutants were further studied by size-exclusion chromatography (SEC).

**Figure 6:**
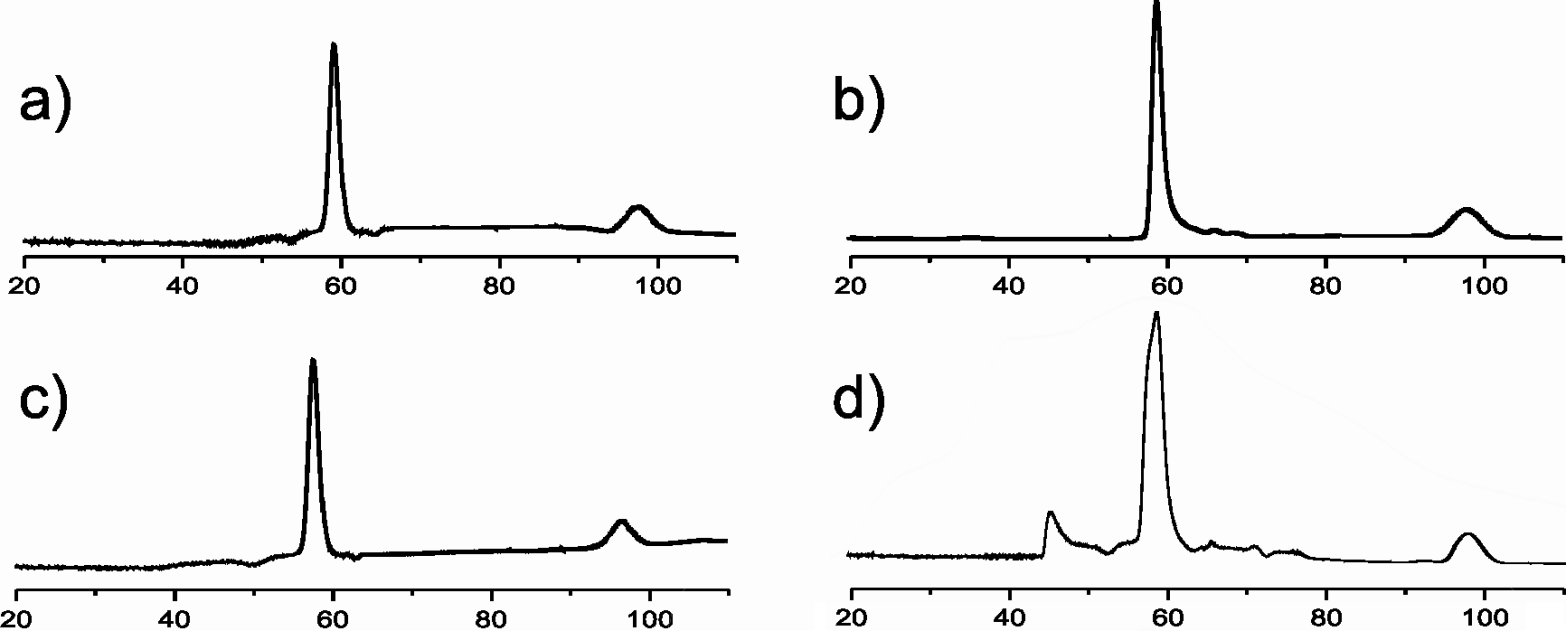
Size-exclusion chromatograms of equimolar mixtures of B3β2γ with (a) Acid-pp, (b) V*^a^*V*^d^*E*^e^*E*^g^*, (c) L*^a^*L*^d^*D*^e^*D*^g^*, and (d) I*^a^*F*^d^*E*^e^*E*^g^* (50 µM of peptide concentration in phosphate buffer 100 mM). The chromatogram of the tetrameric B3β2γ/Acid-pp helix bundle serves as the reference.

The retention-time (*T*_R_*)* value of each mixed system was compared with an equimolar solution of B3β2γ/Acid-pp as a tetrameric reference structure (Figure 6) [18]. The SEC results show that, in analogy to B3β2γ/Acid-pp, tetramers are the predominant oligomerization state in all probed equimolar mixtures of B3β2γ/L*^a^*L*^d^*D*^e^*D*^g^*, B3β2γ/V*^a^*V*^d^*E*^e^*E*^g^*, and B3β2γ/I*^a^*F*^d^*E*^e^*E*^g^* in aqueous solution (59 min). In the case of B3β2γ/I*^a^*F*^d^*E*^e^*E*^g^*, despite the presence of tetrameric helix bundles, other species consistent with a higher order of oligomers can be observed; this has also been indicated by SEC at about 45 min. Whereas the stoichiometry of the interaction on the membrane is considered to be 1:1, the geometry of Phe residues at the hydrophobic core suggests the possibility of aromatic ring stacking in solution. This type of side chain has the distinct feature of being largely indiscriminate in defining a specific oligomerization state; however, it is tolerant of large assemblies [24–25].

Overall, the binding affinities between native mutants and B3β2γ in solution follow the same trends found for the spot intensities on the membrane. The great sequence similarity between synthesized mutants and *wt* sequence allows the study of the impact of new side-chain compositions at *a* and *d* positions.

## Conclusion

A challenge associated with the design of artificial quaternary structures formed by foldameric sequences is to find a suitable α-partner that selectively binds with high affinity. The fact that amino acid side chains exhibit different characteristics depending on the sequence (i.e., structural) context, further complicates the recognition of well-suited side-chain compositions required for a specific interaction between bioactive foldamers and natural targets. This report has presented the application of a simple and sensitive peptide array technique to selectively form stable α-helical coiled-coil structures with an αβγ-chimera. The overall analysis of the interaction between α-partners and αβγ-chimera provides valuable information about the interaction environment accessible to the chimeric motif. Interestingly, small changes (even single mutations) in the sequence of the immobilized α-mutants result in drastic changes in the interaction profile of the αβγ-chimera. Furthermore, this study has identified the residues crucial for forming the recognition epitope of the foldameric βγ-pattern in dependence of the interaction affinities resulting from side-chain mutations in *a* and *d* positions, as well as *e* position of the interacting α-partners. It is also important to note that the coiled-coil pairing selectivity is profoundly increased by bulky hydrophobic side chains at core and core-flanking positions. In terms of binding affinities, the intolerance of these positions to substitution with small or polar amino acids is evidence for the existence of an enlarged interior cavity formed by extended-backbone amino acids, which requires more-space-filling side chains to exclude the surrounding aqueous solution. Finally, these observations suggest that spot technology is an excellent and reliable technique for generating natural sequences that suitably interact with unknown patterns forming artificial coiled-coils.

## Experimental

**SPOT-synthesis** (analogous to a procedure from [26])**:** Cellulose-bound peptide arrays were prepared according to standard SPOT synthesis protocols by using a SPOT synthesizer (Intavis, Köln, Germany) as described in detail in [15]. The peptides were synthesized on amino-functionalized cellulose membranes (Whatman, Maidstone, Great Britain) of the ester type prepared by modifying cellulose paper with Fmoc-β-alanine as the first spacer residue. In the second coupling step, the anchor position Fmoc-β-alanine-OPfp in dimethylsulfoxide (DMSO) was used. Residual amino functions between the spots were capped by acetylation. The Fmoc group was cleaved by using 20% piperidine in dimethylformamide (DMF). The cellulose-bound peptide arrays were assembled on these membranes by using 0.3 M solutions of Fmoc-amino acid-OPfp in 1-methyl-2-pyrrolidone (NMP). The side-chain protection of the Fmoc-amino acids used was as follows: Glu, Asp (O*t*-Bu); Ser, Thr, Tyr (*t*-Bu); His, Lys, Trp (Boc); Asn, Gln, Cys (Trt); Arg (Pbf). After the last coupling step, the acid-labile protection groups of the amino acid side chains were cleaved by using 90% trifluoro-acetic acid (TFA) for 30 min and 60% TFA for 3 h. To ensure adequate quality, the peptides to be analyzed were cleaved from the membrane by using the standard protocol as described by Wenschuh et al. [15] and dissolved in water (using 10% acetonitrile to increase solubility, if necessary). HPLC analysis (Waters, Milford, USA) was conducted by using a linear solvent gradient (A: 0.05% TFA in water; B: 0.05% TFA in acetonitrile; gradient: 5–60% B over 30 min; UV detector at 214 nm; RP-18 column). α-cyanocinnamic acid was used as a matrix for MALDI–TOF (Applied Biosystems, Forster City, USA) MS analysis.

**Binding studies on cellulose membranes** [26]**:** All incubation and washing steps were carried out under gentle shaking and at room temperature. After washing of the membrane with ethanol once for 10 min and three times for 10 min with tris-buffered saline (TBS: 50 mM tris(hydroxymethyl)aminomethane, 137 mM NaCl, 2.7 mM KCl, adjusted to pH 8 with HCl/0.05%), the membrane-bound peptide arrays were blocked (3 h) with blocking buffer (casein-based blocking buffer concentrate (Sigma-Genosys, Cambridge, UK), 1:10 in TBS containing 5% (w/v) sucrose), and then washed with TBS (1 × 10 min). Subsequently, the peptide arrays were incubated with the labeled analytes (*c* = 10 µM) for 10 min in TBS blocking buffer. After washing for 120 min with TBS, analysis and quantification of peptide-bound TAMRA was carried out by using a Lumi-Imager.

**Measurement of spot signal intensities** [26]**:** Binding events (TAMRA-fluorescence) were recorded on a cooled CCD-camera by using a Lumi-Imager (Roche, Indianapolis, USA). The signal intensity (SI) of each spot was calculated by defining a spot radius that can be optimally applied to all spots in the image and taking the median value of the pixel intensity. The background signal was determined with a safety margin to the circular region of each spot, and then the global background mean was subtracted from each individual spot signal. This parameter is referred to as SI. Grid layer and SI were calculated by using dedicated image analysis software: GeneSpotter has a fully automatic grid-finding routine resulting in reproducible signal intensities. The median value of the intraspot distribution was sufficient to avoid saturation. Results are shown as the interspot global background-corrected mean value over three replica spots for each sequence. TAMRA was measured at 645 nm. The aforementioned wavelength was chosen to detect TAMRA at lower background noise.

**Peptide synthesis and characterization** (analogous to [18])**:** Peptides were synthesized by using standard, automated Fmoc solid-phase synthesis (0.05 mM scale) using a SyroXP-I peptide synthesizer (MultiSyn Tech GmbH, Witten, Germany) and HOBT/TBTU activation. Manual coupling of β- and γ-amino acids was carried out by HOAT/DIC activation. The molar excess of amino acid and coupling reagents was reduced for β- and γ-residues to twofold for the first and onefold for the second coupling. The completion of these couplings was indicated by a negative Kaiser test. Prior to each deprotection step, capping of the possibly nonacylated N-termini was carried out by treatment with 10% acetic anhydride and 10% DIEA in DMF (3 × 10 min). Peptide cleavage from resin was performed by using 95% trifluoroacetic acid, 2.5% triisopropylsilane, and 2.5% water. Peptides were purified by HPLC on a C-18 preparative column using gradients between 0.1% TFA in water and 0.1% TFA in acetonitrile. All peptides were >95% pure by analytical HPLC on a C-8 column (Phenomenex^®^ Luna C8, 10 μM, 250 mm × 21.2 mm). The identities of peptides were confirmed using an ESI–TOF instrument.

**Circular-dichroism (CD) spectroscopy** [18]**:** Peptide samples were analyzed in 10 mM phosphate buffer (pH 7.4). Far-ultraviolet circular-dichroism spectra and GndHCl unfolding profiles were recorded on a J-810 spectropolarimeter (Jasco GmbH, Gross-Umstadt, Germany) equipped with a temperature controlled quartz cell of 0.1 cm path length. The recorded spectra were evaluated with the Jasco software package. The spectra were the average of three scans obtained by collecting data from 190–240 nm at 0.2 nm intervals, 2 nm bandwidth, and 1 s response time. Ellipticity data in mdeg were converted to conformation parameters by the following equation: [θ] = [θ]_b_ × mrw/10 × *l* × *c*, where [θ]_b_ is the ellipticity measured in degrees, mrw is the mean residue molecular weight (molecular weight of the peptide divided by the number of amino acid residues), *c* is the peptide concentration in g/mL, and *l* is the optical path length of the cell in cm. Denaturation was carried out in 0.5 °C intervals with a heating rate of 3 °C min^−1^. The midpoints of the thermal melts, *T*_m_s, were taken as the maximum of the derivative d(Fraction unfolded)/d*T*.

**Size-exclusion chromatography (SEC)** [18]**:** The measurements were performed on a VWR-Hitachi Elite LaChrome system (Pump L-2130, UV Detector L-2400, VWR, Germany) equipped with a Superdex 75 PC 3.2/30 column from Amersham Biosciences. The peptides were analyzed in 100 mM sodium phosphate pH 7.4 with a flow rate of 0.025 mL/min. Peptide absorbance was registered at 220 nm. The retention times were corrected with internal and external references. Gly-anthranilic acid was used as an internal reference. GCN4-pLI was employed as a reference for tetrameric coiled coils [27], because its monomer size is comparable to that of the model system used in this study.

## Supporting Information

File 1 Complete set of SPOT intensities.
