# Supplementary material for: Investigation of the network of preferred interactions in an artificial coiled-coil association using the peptide array technique
Source: Beilstein J Org Chem. 2012 Apr 25;8:640–9. doi: 10.3762/bjoc.8.71 (PMC3343290; doi:10.3762/bjoc.8.71)
Supplement: File 1 — Complete set of SPOT intensities. [file Beilstein_J_Org_Chem-08-640-s001.pdf]

Supporting Information

for

Investigation of the network of preferred interactions in an artificial coiled-coil association using the peptide array technique

Raheleh Rezaei Araghi[†1], Carsten C. Mahrenholz[†2], Rudolf Volkmer[2], Beate Koksch*[1]

Address: [1]Institute of Chemistry and Biochemistry, Freie Universität Berlin, Berlin, Germany and [2]Institut für Medizinische Immunologie, Charité-Universitätsmedizin Berlin, Augustenburger Platz 1, 13353, Berlin, Germany.

Email: Beate Koksch* - koksch@chemie.fu-berlin.de

* Corresponding author

[†] These authors contributed equally to this work.

Complete set of SPOT intensities.

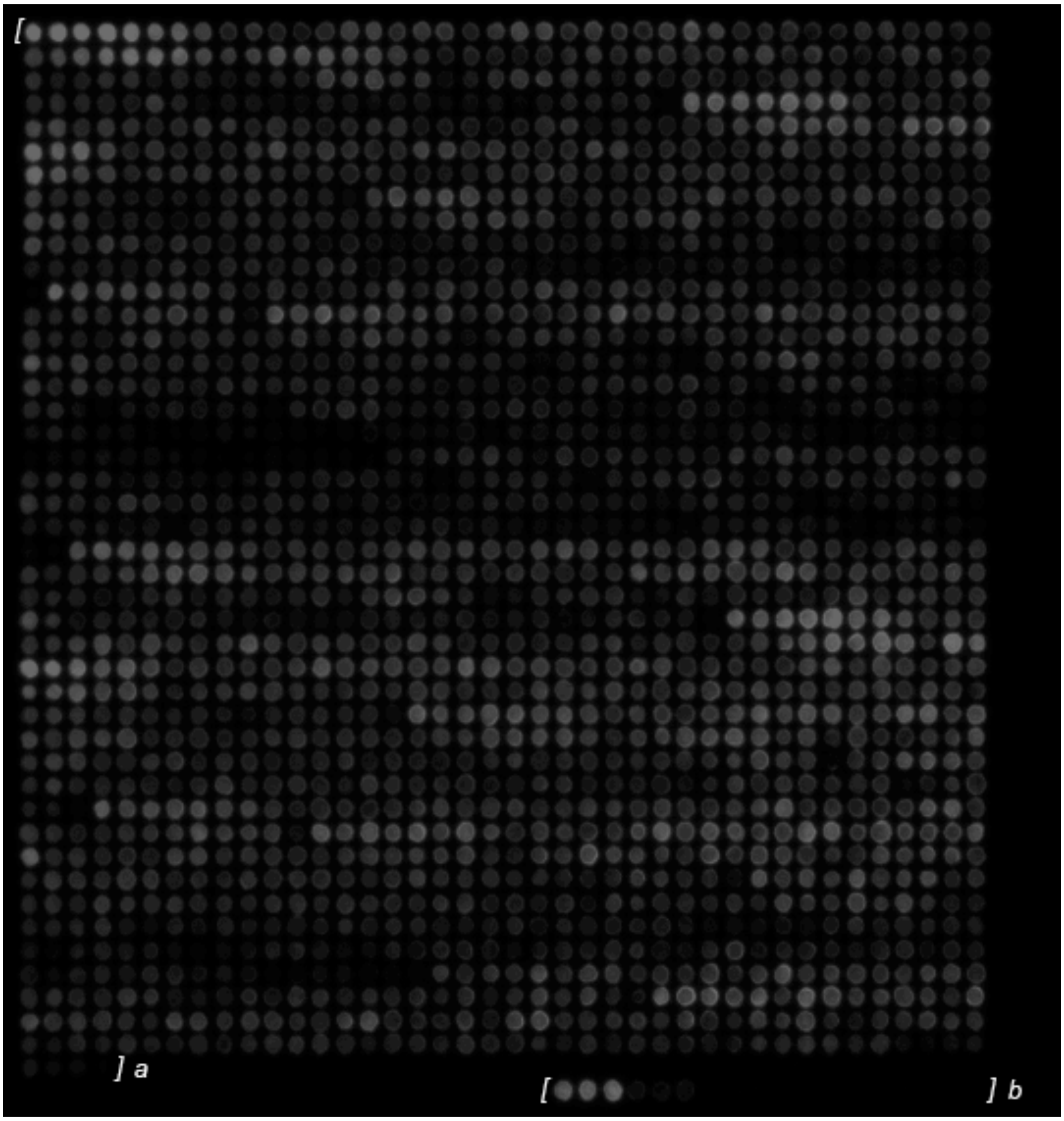

Figure S1. Full set of spot intensities from the membrane probing. a) The random mutation resulting in 1764 spots and (b) the Gly scanning of Acid-pp sequences as described in the main text.

Table S1. The complete set of SPOT intensities detected from 1764 mutants on the cellulose membrane.*

*The values are in Boehringer Light Units (BLUs) and resulted from ($I^{B3\beta2\gamma}$ – $I^{R3\beta2\gamma}$). $I^{B3\beta2\gamma}$ and $I^{RB3\beta2\gamma}$ are the intensity of each spot when the membrane is screened against B3β2γ and R3β2γ, respectively.

LsaLEKE	20424,984	VsaLEKE	7793,704	asaLEKE	4069,144	IsaLEKE	10941,831	FsaLEKE	13737,3	YsaLEKE	11723,013	LsaVEKE	9201,354
LsaLEKV	20796,744	VsaLEKV	11014,524	asaLEKV	4745,624	IsaLEKV	14804,18	FsaLEKV	10274,126	YsaLEKV	7088,457	LsaVEKV	13503,972
LsaLEKL	20395,664	VsaLEKL	10705,409	asaLEKL	6585,222	IsaLEKL	13248,799	FsaLEKL	10674,98	YsaLEKL	9422,829	LsaVEKL	13335,884
LsaLEKI	21117,853	VsaLEKI	12853,853	asaLEKI	9771,788	IsaLEKI	13468,944	FsaLEKI	12110,682	YsaLEKI	6651,75	LsaVEKI	14525,805
LsaLEKa	20557,504	VsaLEKa	10536,255	asaLEKa	9177,914	IsaLEKa	12695,617	FsaLEKa	13086,692	YsaLEKa	12540,664	LsaVEKa	11807,015
LsaLEKY	16438,266	VsaLEKY	7182,462	asaLEKY	4148,947	IsaLEKY	9434,281	FsaLEKY	10553,386	YsaLEKY	6191,737	LsaVEKY	11818,084
LsaLEKD	15830,153	VsaLEKD	8205,8574	asaLEKD	2924,174	IsaLEKD	10051,976	FsaLEKD	12454,004	YsaLEKD	8120,71	LsaVEKD	8407,1209
LsaLVKE	10162,404	VsaLVKE	6282,954	asaLVKE	4812,008	IsaLVKE	7161,744	FsaLVKE	8407,354	YsaLVKE	10166,829	LsaVVKE	6177,856
LsaLVKV	4843,249	VsaLVKV	2715,986	asaLVKV	3196,227	IsaLVKV	3433,79	FsaLVKV	4843,597	YsaLVKV	3829,229	LsaVVKV	2145,268
LsaLVKL	6518,869	VsaLVKL	4811,444	asaLVKL	5760,094	IsaLVKL	4926,65	FsaLVKL	5825,57	YsaLVKL	4506,95	LsaVVKL	4468,524
LsaLVKI	3742,82	VsaLVKI	4209,116	asaLVKI	3467,274	IsaLVKI	4857,994	FsaLVKI	6131,42	YsaLVKI	3964,816	LsaVVKI	3464,219
LsaLVKa	2932,792	VsaLVKa	3961,864	asaLVKa	2984,997	IsaLVKa	3612,185	FsaLVKa	4179,447	YsaLVKa	5425,003	LsaVVKa	1616,364
LsaLVKY	5319,932	VsaLVKY	5366,794	asaLVKY	8224,734	IsaLVKY	4725,81	FsaLVKY	7985,927	YsaLVKY	3113,984	LsaVVKY	3020,757
LsaLVKD	9306,264	VsaLVKD	6042,734	asaLVKD	4394,3906	IsaLVKD	7099,193	FsaLVKD	10704,324	YsaLVKD	7296,222	LsaVVKD	5507,277
LsaLLKE	10129,524	VsaLLKE	10127,363	asaLLKE	7406,012	IsaLLKE	9225,439	FsaLLKE	14055,64	YsaLLKE	5519,539	LsaVLKE	7968,412
LsaLLKV	6318,974	VsaLLKV	4945,477	asaLLKV	4211,594	IsaLLKV	5709,74	FsaLLKV	8936,78	YsaLLKV	4462,944	LsaVLKV	4979,894
LsaLLKL	7851,372	VsaLLKL	6867,994	asaLLKL	4896,454	IsaLLKL	7983,39	FsaLLKL	11792,27	YsaLLKL	5605,612	LsaVLKL	7443,399
LsaLLKI	7323,554	VsaLLKI	5478,528	asaLLKI	4086,334	IsaLLKI	5980,59	FsaLLKI	10848,75	YsaLLKI	2904,76	LsaVLKI	6393,138
LsaLLKa	4552,25	VsaLLKa	4565,734	asaLLKa	3616,614	IsaLLKa	4677,82	FsaLLKa	6515,61	YsaLLKa	3391,832	LsaVLKa	4671,014
LsaLLKY	7697,191	VsaLLKY	6777,474	asaLLKY	2840,034	IsaLLKY	8627,443	FsaLLKY	8785,554	YsaLLKY	2950,076	LsaVLKY	5847,168
LsaLLKD	11299,119	VsaLLKD	8107,807	asaLLKD	5273,742	IsaLLKD	10903,924	FsaLLKD	15064,464	YsaLLKD	5232,893	LsaVLKD	6940,443
LsaLIKE	10774,035	VsaLIKE	7100,329	asaLIKE	4602,996	IsaLIKE	7998,084	FsaLIKE	15128,144	YsaLIKE	5318,624	LsaVIKE	9780,359
LsaLIKV	5886,171	VsaLIKV	3303,976	asaLIKV	3147,376	IsaLIKV	4304,18	FsaLIKV	7224,386	YsaLIKV	3583,021	LsaVIKV	4245,839
LsaLIKL	8638,414	VsaLIKL	5964,631	asaLIKL	4345,673	IsaLIKL	8213,64	FsaLIKL	10971,86	YsaLIKL	4907,914	LsaVIKL	7194,074
LsaLIKI	6498,388	VsaLIKI	3391,41	asaLIKI	3732,266	IsaLIKI	5989,83	FsaLIKI	7832,3	YsaLIKI	4165,34	LsaVIKI	4805,27
LsaLIKa	5138,294	VsaLIKa	2568,863	asaLIKa	3211,634	IsaLIKa	4581,554	FsaLIKa	5655,511	YsaLIKa	4008,206	LsaVIKa	2434,044
LsaLIKY	8183,929	VsaLIKY	10929,711	asaLIKY	4911,244	IsaLIKY	9468,54	FsaLIKY	8551,543	YsaLIKY	3124,014	LsaVIKY	3968,289
LsaLIKD	12451,307	VsaLIKD	9296,917	asaLIKD	3355,768	IsaLIKD	11030,441	FsaLIKD	8909,177	YsaLIKD	5540,051	LsaVIKD	7215,675
LsaLaKE	9553,3516	VsaLaKE	7052,3648	asaLaKE	3304,1789	IsaLaKE	9722,413	FsaLaKE	8560,203	YsaLaKE	5895,684	LsaVaKE	6779,934
LsaLaKV	4229,794	VsaLaKV	2549,774	asaLaKV	1881,446	IsaLaKV	4232,615	FsaLaKV	3429,052	YsaLaKV	2383,648	LsaVaKV	2191,854
LsaLaKL	4902,384	VsaLaKL	3269,8678	asaLaKL	2370,154	IsaLaKL	6134,668	FsaLaKL	4045,489	YsaLaKL	3181,834	LsaVaKL	3250,364
LsaLaKI	5317,494	VsaLaKI	2887,932	asaLaKI	2442,259	IsaLaKI	5102,63	FsaLaKI	2997,93	YsaLaKI	1994,304	LsaVaKI	6085,374
LsaLaKa	2644,5285	VsaLaKa	984,955	asaLaKa	2104,9944	IsaLaKa	3298,884	FsaLaKa	2900,564	YsaLaKa	4325,189	LsaVaKa	2978,374
LsaLaKY	3842,829	VsaLaKY	2997,437	asaLaKY	1901,371	IsaLaKY	4394,971	FsaLaKY	3674,971	YsaLaKY	1389,455	LsaVaKY	3863,655
LsaLaKD	5891,7987	VsaLaKD	4835,067	asaLaKD	2615,1658	IsaLaKD	7918,454	FsaLaKD	5814,01	YsaLaKD	6659,961	LsaVaKD	6073,364
LsaLYKE	6647,009	VsaLYKE	5431,053	asaLYKE	4857,384	IsaLYKE	7789,262	FsaLYKE	6334,47	YsaLYKE	4374,409	LsaVYKE	6093,696
LsaLYKV	4220,864	VsaLYKV	3535,707	asaLYKV	3849,764	IsaLYKV	3822,294	FsaLYKV	3454,624	YsaLYKV	2583,11	LsaVYKV	2299,362
LsaLYKL	5388,24	VsaLYKL	4653,056	asaLYKL	3794,654	IsaLYKL	4848,941	FsaLYKL	5006,024	YsaLYKL	3826,586	LsaVYKL	4369,769
LsaLYKI	3575,479	VsaLYKI	4053,422	asaLYKI	4187,036	IsaLYKI	5520,835	FsaLYKI	4544,495	YsaLYKI	2917,084	LsaVYKI	3228,488
LsaLYKa	2567,102	VsaLYKa	3335,687	asaLYKa	5038,552	IsaLYKa	3399,536	FsaLYKa	3613,901	YsaLYKa	4829,305	LsaVYKa	2131,099
LsaLYKY	8779,764	VsaLYKY	3003,564	asaLYKY	2318,624	IsaLYKY	4689,41	FsaLYKY	3919,934	YsaLYKY	3109,945	LsaVYKY	2906,013
LsaLYKD	10396,715	VsaLYKD	4836,893	asaLYKD	3574,189	IsaLYKD	6324,617	FsaLYKD	6823,248	YsaLYKD	9412,226	LsaVYKD	4394,996
LsaLDKE	14325,184	VsaLDKE	4954,1107	asaLDKE	984,0225	IsaLDKE	8630,939	FsaLDKE	6755,755	YsaLDKE	6654,514	LsaVDKE	3935,014
LsaLDKV	17048,023	VsaLDKV	8494,002	asaLDKV	4220,569	IsaLDKV	11575,564	FsaLDKV	9186,417	YsaLDKV	5515,368	LsaVDKV	9167,295
LsaLDKL	19113,994	VsaLDKL	8793,145	asaLDKL	5388,204	IsaLDKL	13975,966	FsaLDKL	9940,455	YsaLDKL	9129,626	LsaVDKL	9382,575
LsaLDKI	18424,553	VsaLDKI	9634,814	asaLDKI	4512,106	IsaLDKI	13148,805	FsaLDKI	10509,664	YsaLDKI	6011,856	LsaVDKI	10106,555
LsaLDKa	16391,066	VsaLDKa	8203,807	asaLDKa	4573,015	IsaLDKa	11419,427	FsaLDKa	10510,584	YsaLDKa	9893,357	LsaVDKa	6724,222
LsaLDKY	10789,644	VsaLDKY	7065,764	asaLDKY	2574,261	IsaLDKY	9920,58	FsaLDKY	7626,907	YsaLDKY	5099,866	LsaVDKY	7697,334
LsaLDKD	7568,504	VsaLDKD	2895,8136	asaLDKD	296,9105	IsaLDKD	6460,344	FsaLDKD	4429,046	YsaLDKD	3018,184	LsaVDKD	1569,934
LsaVEKE	9201,354	VsaVEKE	4487,474	asaVEKE	189,5977	IsaVEKE	7748,494	FsaVEKE	7686,152	YsaVEKE	2704,179		
LsaVEKV	13503,972	VsaVEKV	5978,094	asaVEKV	1950,334	IsaVEKV	7672,317	FsaVEKV	8782,872	YsaVEKV	2968,514		
LsaVEKL	13335,884	VsaVEKL	8030,519	asaVEKL	2979,801	IsaVEKL	8704,574	FsaVEKL	10264,68	YsaVEKL	3841,314		
LsaVEKI	14525,805	VsaVEKI	6922,985	asaVEKI	4915,6968	IsaVEKI	8307,985	FsaVEKI	11243,104	YsaVEKI	3945,6179		down:
LsaVEKa	11807,015	VsaVEKa	9592,644	asaVEKa	4953,66	IsaVEKa	10616,155	FsaVEKa	13427,553	YsaVEKa	5485,714		right
LsaVEKY	11818,084	VsaVEKY	4647,518	asaVEKY	1566,614	IsaVEKY	4744,914	FsaVEKY	10993,144	YsaVEKY	2436,61		
LsaVEKD	8407,1209	VsaVEKD	3111,2811	asaVEKD	-16,656	IsaVEKD	6137,5648	FsaVEKD	6562,457	YsaVEKD	708,8962		
LsaVVKE	6177,856	VsaVVKE	5186,891	asaVVKE	2473,6917	IsaVVKE	5890,733	FsaVVKE	8608,65	YsaVVKE	2709,206		
LsaVVKV	2145,268	VsaVVKV	2124,235	asaVVKV	1874,324	IsaVVKV	2857,51	FsaVVKV	3430,694	YsaVVKV	1110,837		
LsaVVKL	4468,524	VsaVVKL	4334,764	asaVVKL	2855,997	IsaVVKL	5093,4	FsaVVKL	5647,834	YsaVVKL	2953,939		
LsaVVKI	3464,219	VsaVVKI	3446,56	asaVVKI	1801,916	IsaVVKI	4305,26	FsaVVKI	4323,088	YsaVVKI	2115,394		

LsaVVKa 1616,364	VsaVVKa 1659,114	asaVVKa 2129,032	IsaVVKa 3060,617	FsaVVKa 3639,564	YsaVVKa 2192,882
LsaVVKY 3020,757	VsaVVKY 1975,231	asaVVKY 1844,162	IsaVVKY 3542,754	FsaVVKY 5943,552	YsaVVKY 1836,707
LsaVVKD 5507,277	VsaVVKD 3228,092	asaVVKD 110,595	IsaVVKD 4908,259	FsaVVKD 7256,534	YsaVVKD 2566,057
LsaVLKE 7968,412	VsaVLKE 7806,195	asaVLKE 3745,379	IsaVLKE 11424,144	FsaVLKE 11219,217	YsaVLKE 4271,752
LsaVLKV 4979,894	VsaVLKV 3595,714	asaVLKV 3267,548	IsaVLKV 6906,984	FsaVLKV 4966,666	YsaVLKV 2428,619
LsaVLKL 7443,399	VsaVLKL 6194,63	asaVLKL 5147,875	IsaVLKL 9449,55	FsaVLKL 7538,884	YsaVLKL 3398,403
LsaVLKI 6393,138	VsaVLKI 9552,072	asaVLKI 4429,584	IsaVLKI 8829,09	FsaVLKI 5282,52	YsaVLKI 2358,21
LsaVLKa 4671,014	VsaVLKa 6561,914	asaVLKa 2159,537	IsaVLKa 7854,923	FsaVLKa 3604,326	YsaVLKa 1683,522
LsaVLKY 5847,168	VsaVLKY 5086,176	asaVLKY 2207,284	IsaVLKY 7717,21	FsaVLKY 2984,694	YsaVLKY 1450,188
LsaVLKD 6940,443	VsaVLKD 8245,326	asaVLKD 2653,6387	IsaVLKD 15106,684	FsaVLKD 5867,304	YsaVLKD 2811,824
LsaVIKE 9780,359	VsaVIKE 6276,084	asaVIKE 3384,488	IsaVIKE 10793,011	FsaVIKE 5803,227	YsaVIKE 4001,839
LsaVIKV 4245,839	VsaVIKV 3054,139	asaVIKV 2571,504	IsaVIKV 4717,33	FsaVIKV 2384,375	YsaVIKV 1077,616
LsaVIKL 7194,074	VsaVIKL 5617,166	asaVIKL 4234,419	IsaVIKL 6938,65	FsaVIKL 4561,614	YsaVIKL 3165,414
LsaVIKI 4805,27	VsaVIKI 3503,177	asaVIKI 3261,594	IsaVIKI 5954,39	FsaVIKI 3505,89	YsaVIKI 1807,432
LsaVIKa 2434,044	VsaVIKa 2173,216	asaVIKa 2655,594	IsaVIKa 5480,785	FsaVIKa 2149,846	YsaVIKa 2170,398
LsaVIKY 3968,289	VsaVIKY 4483,234	asaVIKY 2411,202	IsaVIKY 5842,075	FsaVIKY 2895,744	YsaVIKY 2545,272
LsaVIKD 7215,675	VsaVIKD 4571,475	asaVIKD 1634,3046	IsaVIKD 9662,544	FsaVIKD 3922,765	YsaVIKD 3048,868
LsaVaKE 6779,934	VsaVaKE 4467,804	asaVaKE 987,834	IsaVaKE 6183,814	FsaVaKE 5484,618	YsaVaKE 1888,004
LsaVaKV 2191,854	VsaVaKV 1020,454	asaVaKV 1334,477	IsaVaKV 3905,205	FsaVaKV 2114,04	YsaVaKV 338,773
LsaVaKL 3250,364	VsaVaKL 2328,345	asaVaKL 3265,451	IsaVaKL 3680,492	FsaVaKL 3407,95	YsaVaKL 457,396
LsaVaKI 6085,374	VsaVaKI 2017,172	asaVaKI 2139,066	IsaVaKI 3392,602	FsaVaKI 3231,77	YsaVaKI 964,774
LsaVaKa 2978,374	VsaVaKa 772,238	asaVaKa 1444,454	IsaVaKa 1436,185	FsaVaKa 2230,234	YsaVaKa 1616,386
LsaVaKY 3863,655	VsaVaKY 1354,83	asaVaKY 870,971	IsaVaKY 2211,074	FsaVaKY 2612,176	YsaVaKY 193,007
LsaVaKD 6073,364	VsaVaKD 2441,263	asaVaKD 199,6229	IsaVaKD 4550,2886	FsaVaKD 4127,155	YsaVaKD 3033,888
LsaVYKE 6093,696	VsaVYKE 4406,834	asaVYKE 1839,181	IsaVYKE 6388,534	FsaVYKE 5835,854	YsaVYKE 4318,03
LsaVYKV 2299,362	VsaVYKV 2300,19	asaVYKV 1772,364	IsaVYKV 1927,124	FsaVYKV 2768,943	YsaVYKV 2253,528
LsaVYKL 4369,769	VsaVYKL 3242,91	asaVYKL 2881,183	IsaVYKL 4054,946	FsaVYKL 4079,074	YsaVYKL 2799,426
LsaVYKI 3228,488	VsaVYKI 2685,224	asaVYKI 2565,338	IsaVYKI 3369,736	FsaVYKI 3750,874	YsaVYKI 3061,934
LsaVYKa 2131,099	VsaVYKa 2090,6	asaVYKa 2004,002	IsaVYKa 1878,834	FsaVYKa 3839,333	YsaVYKa 2840,742
LsaVYKY 2906,013	VsaVYKY 2820,744	asaVYKY 1671,744	IsaVYKY 3088,105	FsaVYKY 3648,864	YsaVYKY 1789,28
LsaVYKD 4394,996	VsaVYKD 2596,918	asaVYKD -91,5958	IsaVYKD 5045,938	FsaVYKD 4400,762	YsaVYKD 1788,2817
LsaVDKE 3935,014	VsaVDKE 57,9561	asaVDKE -40,056	IsaVDKE 2260,123	FsaVDKE 4309,684	YsaVDKE 153,3274
LsaVDKV 9167,295	VsaVDKV 3435,4957	asaVDKV 1660,29	IsaVDKV 6125,994	FsaVDKV 5192,054	YsaVDKV 2433,845
LsaVDKL 9382,575	VsaVDKL 5039,762	asaVDKL 3482,194	IsaVDKL 9072,613	FsaVDKL 7065,223	YsaVDKL 3213,954
LsaVDKI 10106,555	VsaVDKI 3075,664	asaVDKI 1887,004	IsaVDKI 8310,208	FsaVDKI 9940,546	YsaVDKI 2678,954
LsaVDKa 6724,222	VsaVDKa 3580,8764	asaVDKa 804,397	IsaVDKa 6969,47	FsaVDKa 7523,314	YsaVDKa 2525,909
LsaVDKY 7697,334	VsaVDKY 3741,971	asaVDKY 943,7446	IsaVDKY 4079,144	FsaVDKY 5725,034	YsaVDKY 1053,353
LsaVDKD 1569,934	VsaVDKD -40,9003	asaVDKD 0	IsaVDKD 749,279	FsaVDKD 539,778	YsaVDKD -30,039
LsaaEKE 3236,2825	VsaaEKE 1324,0491	asaaEKE 0	IsaaEKE 1791,734	FsaaEKE 4827,463	YsaaEKE 964,243
LsaaEKV 6025,4679	VsaaEKV 3081,804	asaaEKV -18,9028	IsaaEKV 3884,454	FsaaEKV 6530,583	YsaaEKV 1471,7099
LsaaEKL 7760,354	VsaaEKL 3599,004	asaaEKL 917,4092	IsaaEKL 4679,182	FsaaEKL 11422,701	YsaaEKL 2955,664
LsaaEKI 8424,374	VsaaEKI 2839,514	asaaEKI 375,865	IsaaEKI 4798,085	FsaaEKI 11920,716	YsaaEKI 3617,9075
LsaaEKa 6666,084	VsaaEKa 3401,3415	asaaEKa 164,6163	IsaaEKa 5311,256	FsaaEKa 10535,984	YsaaEKa 2200,985
LsaaEKY 5762,089	VsaaEKY 1721,178	asaaEKY -17,7805	IsaaEKY 2109,114	FsaaEKY 6351,644	YsaaEKY 603,048
LsaaEKD 2989,9411	VsaaEKD 16,2504	asaaEKD -48,1748	IsaaEKD 1699,3515	FsaaEKD 4220,12	YsaaEKD 1197,3648
LsaaVKE 6827,638	VsaaVKE 3089,414	asaaVKE -84,14123	IsaaVKE 4754,841	FsaaVKE 7041,924	YsaaVKE 3121,914
LsaaVKV 2640,524	VsaaVKV 2793,534	asaaVKV 394,671	IsaaVKV 1516,154	FsaaVKV 3478,615	YsaaVKV 1432,594
LsaaVKL 4530,446	VsaaVKL 3543,051	asaaVKL 1064,596	IsaaVKL 3342,327	FsaaVKL 3899,792	YsaaVKL 3626,494
LsaaVKI 3159,356	VsaaVKI 3099,064	asaaVKI 820,014	IsaaVKI 3495,404	FsaaVKI 4122,576	YsaaVKI 2718,832
LsaaVKa 2115,844	VsaaVKa 3402,239	asaaVKa 933,349	IsaaVKa 3729,448	FsaaVKa 3290,49	YsaaVKa 1420,556
LsaaVKY 3407,482	VsaaVKY 2327,513	asaaVKY 1560,265	IsaaVKY 2980,304	FsaaVKY 1434,83	YsaaVKY 398,755
LsaaVKD 6711,1068	VsaaVKD 4068,9886	asaaVKD -103,52	IsaaVKD 4899,74	FsaaVKD 4691,454	YsaaVKD 2391,652
LsaaLKE 7778,05	VsaaLKE 5860,809	asaaLKE 1704,5595	IsaaLKE 9556,079	FsaaLKE 7261,034	YsaaLKE 3112,634
LsaaLKV 3924,14	VsaaLKV 2615,806	asaaLKV 1631,931	IsaaLKV 4179,811	FsaaLKV 3561,477	YsaaLKV 2545,65
LsaaLKL 4608,289	VsaaLKL 4394,308	asaaLKL 2839,379	IsaaLKL 7187,649	FsaaLKL 3849,684	YsaaLKL 3235,624
LsaaLKI 3517,744	VsaaLKI 4190,095	asaaLKI 2426,927	IsaaLKI 6219,351	FsaaLKI 4738,215	YsaaLKI 4251,094
LsaaLKa 2573,5263	VsaaLKa 2969,478	asaaLKa 1739,682	IsaaLKa 4777,748	FsaaLKa 3194,797	YsaaLKa 1297,611
LsaaLKY 3815,744	VsaaLKY 3610,484	asaaLKY 1751,114	IsaaLKY 5596,476	FsaaLKY 1956,01	YsaaLKY 1284,824
LsaaLKD 7701,425	VsaaLKD 5682,274	asaaLKD 835,7793	IsaaLKD 10961,1924	FsaaLKD 6552,594	YsaaLKD 4181,334
LsaaIKE 8454,374	VsaaIKE 5392,811	asaaIKE 1947,5746	IsaaIKE 5633,328	FsaaIKE 4987,454	YsaaIKE 6250,154
LsaaIKV 4916,2925	VsaaIKV 935,038	asaaIKV 873,649	IsaaIKV 1761,018	FsaaIKV 3131,636	YsaaIKV 1696,214

LsaaIKL	5212,844	VsaaIKL	2074,044	asaaIKL	2216,674	IsaaIKL	3203,214	FsaaIKL	3526,245	YsaaIKL	2976,941
LsaaIKI	4378,904	VsaaIKI	2422,174	asaaIKI	2255,309	IsaaIKI	3236,124	FsaaIKI	3354,464	YsaaIKI	3068,174
LsaaIKa	3999,614	VsaaIKa	2576,505	asaaIKa	1665,754	IsaaIKa	2672,998	FsaaIKa	2150,453	YsaaIKa	2574,438
LsaaIKY	3251,06	VsaaIKY	1273,539	asaaIKY	1376,593	IsaaIKY	2202,98	FsaaIKY	1996,807	YsaaIKY	1661,714
LsaaIKD	8366,9068	VsaaIKD	5014,314	asaaIKD	97,3051	IsaaIKD	4322,924	FsaaIKD	5551,734	YsaaIKD	3418,986
LsaaaKE	4406,5466	VsaaaKE	1580,974	asaaaKE	-65,6062	IsaaaKE	1929,557	FsaaaKE	5330,8065	YsaaaKE	3949,024
LsaaaKV	1321,6944	VsaaaKV	381,011	asaaaKV	348,5081	IsaaaKV	531,915	FsaaaKV	2644,167	YsaaaKV	1303,854
LsaaaKL	2337,261	VsaaaKL	490,073	asaaaKL	966,584	IsaaaKL	1830,294	FsaaaKL	3364,584	YsaaaKL	2852,547
LsaaaKI	2479,2693	VsaaaKI	700,123	asaaaKI	374,014	IsaaaKI	987,59	FsaaaKI	3091,713	YsaaaKI	2039,514
LsaaaKa	783,167	VsaaaKa	239,025	asaaaKa	-79,9628	IsaaaKa	887,696	FsaaaKa	2955,52	YsaaaKa	1622,154
LsaaaKY	804,203	VsaaaKY	511,557	asaaaKY	-154,504	IsaaaKY	627,575	FsaaaKY	1615,39	YsaaaKY	492,737
LsaaaKD	3339,7382	VsaaaKD	1595,184	asaaaKD	0	IsaaaKD	2463,6548	FsaaaKD	3606,664	YsaaaKD	3041,3807
LsaaYKE	2626,246	VsaaYKE	1846,634	asaaYKE	526,3246	IsaaYKE	2420,844	FsaaYKE	7215,914	YsaaYKE	3142,344
LsaaYKV	1070,699	VsaaYKV	1998,251	asaaYKV	816,2305	IsaaYKV	2580,118	FsaaYKV	3999,014	YsaaYKV	2285,663
LsaaYKL	2827,464	VsaaYKL	2855,555	asaaYKL	1643,2202	IsaaYKL	2901,747	FsaaYKL	6157,387	YsaaYKL	3837,014
LsaaYKI	2648,787	VsaaYKI	2405,809	asaaYKI	1099,484	IsaaYKI	2739,794	FsaaYKI	4962,96	YsaaYKI	3231,144
LsaaYKa	873,8396	VsaaYKa	2314,309	asaaYKa	934,5601	IsaaYKa	1839,891	FsaaYKa	3951,95	YsaaYKa	1484,206
LsaaYKY	1522,402	VsaaYKY	2783,463	asaaYKY	270,366	IsaaYKY	2001,52	FsaaYKY	3730,275	YsaaYKY	1538,037
LsaaYKD	1995,0242	VsaaYKD	1831,0085	asaaYKD	-20,0139	IsaaYKD	2973,233	FsaaYKD	5133,528	YsaaYKD	1412,308
LsaaDKE	485,5395	VsaaDKE	-53,876	asaaDKE	0	IsaaDKE	494,5022	FsaaDKE	527,2297	YsaaDKE	-52,1552
LsaaDKV	2527,0678	VsaaDKV	592,1047	asaaDKV	-33,0767	IsaaDKV	1980,802	FsaaDKV	2471,954	YsaaDKV	34,1016
LsaaDKL	4346,3438	VsaaDKL	1548,171	asaaDKL	233,203	IsaaDKL	4176,127	FsaaDKL	5521,632	YsaaDKL	1325,164
LsaaDKI	4422,664	VsaaDKI	1254,242	asaaDKI	14,9909	IsaaDKI	3996,854	FsaaDKI	4027,019	YsaaDKI	1114,354
LsaaDKa	3623,6954	VsaaDKa	359,098	asaaDKa	-11,8022	IsaaDKa	2949,8668	FsaaDKa	3083,214	YsaaDKa	-66,376
LsaaDKY	4010,1515	VsaaDKY	522,695	asaaDKY	-102,036	IsaaDKY	2179,38	FsaaDKY	2263,1963	YsaaDKY	-112,545
LsaaDKD	-11,0521	VsaaDKD	0	asaaDKD	-11,1368	IsaaDKD	-61,4989	FsaaDKD	0	YsaaDKD	-98,4689
LsaIEKE	15737,724	VsaIEKE	15205,1723	asaIEKE	2534,6982	IsaIEKE	14911,99	FsaIEKE	13578,162	YsaIEKE	6629,298
LsaIEKV	16170,453	VsaIEKV	12042,142	asaIEKV	3080,9885	IsaIEKV	13666,245	FsaIEKV	9653,495	YsaIEKV	3576,743
LsaIEKL	16125,888	VsaIEKL	11854,844	asaIEKL	4411,674	IsaIEKL	17489,46	FsaIEKL	11145,533	YsaIEKL	6381,214
LsaIEKI	17376,151	VsaIEKI	11241,922	asaIEKI	4262,889	IsaIEKI	17353,992	FsaIEKI	10378,838	YsaIEKI	5272,411
LsaIEKa	18089,619	VsaIEKa	11139,764	asaIEKa	6101,814	IsaIEKa	19135,443	FsaIEKa	11946,339	YsaIEKa	10716,827
LsaIEKY	15177,559	VsaIEKY	8171,991	asaIEKY	3279,754	IsaIEKY	14555,242	FsaIEKY	9080	YsaIEKY	5040,872
LsaIEKD	17113,264	VsaIEKD	8732,4102	asaIEKD	1621,874	IsaIEKD	16191,1	FsaIEKD	9602,814	YsaIEKD	8404,054
LsaIVKE	6877,351	VsaIVKE	5738,794	asaIVKE	4533,044	IsaIVKE	11227,446	FsaIVKE	5722,472	YsaIVKE	7932,274
LsaIVKV	3310,68	VsaIVKV	2732,39	asaIVKV	2670,158	IsaIVKV	5204,139	FsaIVKV	3036,509	YsaIVKV	3623,362
LsaIVKL	5264,944	VsaIVKL	5533,443	asaIVKL	4438,099	IsaIVKL	6886,12	FsaIVKL	3746,69	YsaIVKL	4299,666
LsaIVKI	4214,994	VsaIVKI	3360,554	asaIVKI	3272,184	IsaIVKI	5198,7	FsaIVKI	2893,92	YsaIVKI	3690,656
LsaIVKa	2768,953	VsaIVKa	2825,316	asaIVKa	2455,71	IsaIVKa	5804,328	FsaIVKa	2979,778	YsaIVKa	7126,43
LsaIVKY	4553,882	VsaIVKY	3722,676	asaIVKY	3504,839	IsaIVKY	5412,195	FsaIVKY	4096,956	YsaIVKY	3656,72
LsaIVKD	10782,424	VsaIVKD	5104,104	asaIVKD	2550,199	IsaIVKD	8753,827	FsaIVKD	6970,93	YsaIVKD	7424,833
LsaILKE	10706,792	VsaILKE	7967,848	asaILKE	6565,982	IsaILKE	10944,845	FsaILKE	7360,162	YsaILKE	12225,964
LsaILKV	5431,074	VsaILKV	3328,003	asaILKV	4205,02	IsaILKV	5133,356	FsaILKV	4657,192	YsaILKV	5340,96
LsaILKL	7530,894	VsaILKL	6971,667	asaILKL	7096,03	IsaILKL	7895,17	FsaILKL	3529,22	YsaILKL	6305,62
LsaILKI	5932,754	VsaILKI	4040,061	asaILKI	4645,25	IsaILKI	4930,02	FsaILKI	6263,996	YsaILKI	5795,99
LsaILKa	4172,756	VsaILKa	3555,768	asaILKa	3786,915	IsaILKa	3039,36	FsaILKa	3611,987	YsaILKa	5548,261
LsaILKY	5156,741	VsaILKY	4939,695	asaILKY	3643,89	IsaILKY	6656,494	FsaILKY	4983,136	YsaILKY	3908,744
LsaILKD	7800,839	VsaILKD	9036,886	asaILKD	5592,214	IsaILKD	12768,147	FsaILKD	7504,814	YsaILKD	8192,679
LsaIIKE	6803,886	VsaIIKE	7076,054	asaIIKE	6244,79	IsaIIKE	7461,911	FsaIIKE	7248,117	YsaIIKE	7332,904
LsaIIKV	4027,779	VsaIIKV	3557,153	asaIIKV	3550,634	IsaIIKV	3970,56	FsaIIKV	4801,014	YsaIIKV	4102,768
LsaIIKL	5738,034	VsaIIKL	6755,574	asaIIKL	5284,531	IsaIIKL	6755,5	FsaIIKL	6863,41	YsaIIKL	4214,884
LsaIIKI	4457,02	VsaIIKI	5017,614	asaIIKI	3856,052	IsaIIKI	4791,55	FsaIIKI	5605,814	YsaIIKI	5232,545
LsaIIKa	3031,534	VsaIIKa	3229,867	asaIIKa	4270,864	IsaIIKa	3871,511	FsaIIKa	5149,563	YsaIIKa	3856,079
LsaIIKY	4516,207	VsaIIKY	4189,032	asaIIKY	4275,232	IsaIIKY	5078,45	FsaIIKY	6838,683	YsaIIKY	3269,936
LsaIIKD	6250,964	VsaIIKD	6760,955	asaIIKD	4541,744	IsaIIKD	9202,414	FsaIIKD	9553,291	YsaIIKD	6789,19
LsaIaKE	6251,966	VsaIaKE	5892,714	asaIaKE	3688,4187	IsaIaKE	7856,54	FsaIaKE	13354,753	YsaIaKE	5550,178
LsaIaKV	2404,766	VsaIaKV	2402,462	asaIaKV	1468,955	IsaIaKV	2346,52	FsaIaKV	5042,669	YsaIaKV	1120,195
LsaIaKL	3388,214	VsaIaKL	3692,984	asaIaKL	2193,742	IsaIaKL	3126,962	FsaIaKL	6302,734	YsaIaKL	1854,024
LsaIaKI	3149,094	VsaIaKI	2983,758	asaIaKI	1444,552	IsaIaKI	3625,815	FsaIaKI	4301,714	YsaIaKI	1894,994
LsaIaKa	1718,2587	VsaIaKa	2981,704	asaIaKa	1246,024	IsaIaKa	3647,794	FsaIaKa	5306,922	YsaIaKa	4327,584
LsaIaKY	3722,57	VsaIaKY	1456,943	asaIaKY	1304,684	IsaIaKY	3078,259	FsaIaKY	6242,839	YsaIaKY	2167,382
LsaIaKD	5603,8207	VsaIaKD	4664,084	asaIaKD	1726,841	IsaIaKD	6422,444	FsaIaKD	12378,004	YsaIaKD	5133,426

LsaIYKE	6979,594	VsaIYKE	5037,25	asaIYKE	3804,147	IsaIYKE	7151,254	FsaIYKE	13334,624	YsaIYKE	4805,16
LsaIYKV	2240,534	VsaIYKV	2531,814	asaIYKV	2234,439	IsaIYKV	2708,192	FsaIYKV	4599,774	YsaIYKV	2757,303
LsaIYKL	3474,937	VsaIYKL	3462,194	asaIYKL	2660,614	IsaIYKL	4376,558	FsaIYKL	6754,884	YsaIYKL	4602,887
LsaIYKI	3688,716	VsaIYKI	2331,318	asaIYKI	3202,865	IsaIYKI	3862,344	FsaIYKI	4955,55	YsaIYKI	3341,034
LsaIYKa	2586,294	VsaIYKa	5081,992	asaIYKa	2749,494	IsaIYKa	2850,893	FsaIYKa	3071,394	YsaIYKa	4597,176
LsaIYKY	3643,874	VsaIYKY	4169,763	asaIYKY	2116,24	IsaIYKY	4022,633	FsaIYKY	3557,409	YsaIYKY	2591,563
LsaIYKD	6758,076	VsaIYKD	6490,236	asaIYKD	1638,6109	IsaIYKD	7668,354	FsaIYKD	5880,327	YsaIYKD	5574,254
LsaIDKE	9171,424	VsaIDKE	5185,074	asaIDKE	198,31242	IsaIDKE	10835,633	FsaIDKE	6022,975	YsaIDKE	2041,644
LsaIDKV	11944,509	VsaIDKV	7540,517	asaIDKV	2446,078	IsaIDKV	13298,764	FsaIDKV	7420,037	YsaIDKV	4672,597
LsaIDKL	13020,73	VsaIDKL	9010,36	asaIDKL	3624,674	IsaIDKL	18077,202	FsaIDKL	11793,93	YsaIDKL	8405,311
LsaIDKI	11636,684	VsaIDKI	8105,846	asaIDKI	3523,07	IsaIDKI	14530,429	FsaIDKI	7746,849	YsaIDKI	4978,035
LsaIDKa	12727,453	VsaIDKa	6236,676	asaIDKa	3480,961	IsaIDKa	17607,153	FsaIDKa	7928,206	YsaIDKa	8411,434
LsaIDKY	10492,199	VsaIDKY	7255,837	asaIDKY	2071,594	IsaIDKY	14531,772	FsaIDKY	6978,104	YsaIDKY	3490,374
LsaIDKD	5090,4342	VsaIDKD	1625,784	asaIDKD	67,0296	IsaIDKD	5898,464	FsaIDKD	1839,484	YsaIDKD	1890,313
LsaFEKE	15490,304	VsaFEKE	13385,7636	asaFEKE	3175,3204	IsaFEKE	20267,119	FsaFEKE	13363,576	YsaFEKE	13616,249
LsaFEKV	12971,696	VsaFEKV	10571,592	asaFEKV	3801,884	IsaFEKV	15234,264	FsaFEKV	9450,356	YsaFEKV	11828,511
LsaFEKL	13864,892	VsaFEKL	14296,98	asaFEKL	5172,273	IsaFEKL	19554,113	FsaFEKL	12218,664	YsaFEKL	13013,57
LsaFEKI	12352,794	VsaFEKI	10479,251	asaFEKI	5509,676	IsaFEKI	18196,984	FsaFEKI	10244,888	YsaFEKI	9062,193
LsaFEKa	19418,144	VsaFEKa	12403,81	asaFEKa	6359,394	IsaFEKa	17598,286	FsaFEKa	13776,672	YsaFEKa	13289,814
LsaFEKY	15526,753	VsaFEKY	8907,764	asaFEKY	3155,067	IsaFEKY	12742,184	FsaFEKY	8587,074	YsaFEKY	5464,744
LsaFEKD	17616,184	VsaFEKD	9143,0482	asaFEKD	1706,894	IsaFEKD	14358,176	FsaFEKD	12999,2	YsaFEKD	14684,142
LsaFVKE	10566,084	VsaFVKE	9081,984	asaFVKE	5049,96	IsaFVKE	9538,147	FsaFVKE	8320,307	YsaFVKE	10314,926
LsaFVKV	4449,603	VsaFVKV	4319,631	asaFVKV	2817,265	IsaFVKV	3275,51	FsaFVKV	3892,964	YsaFVKV	6232,96
LsaFVKL	6250,221	VsaFVKL	6275,851	asaFVKL	5524,748	IsaFVKL	6242,639	FsaFVKL	6154,636	YsaFVKL	6690,59
LsaFVKI	4509,661	VsaFVKI	5511,917	asaFVKI	3221,034	IsaFVKI	4969,96	FsaFVKI	6466,46	YsaFVKI	6754,292
LsaFVKa	3224,932	VsaFVKa	3890,654	asaFVKa	3154,874	IsaFVKa	4950,179	FsaFVKa	5200,851	YsaFVKa	6650,594
LsaFVKY	4162,023	VsaFVKY	5148,208	asaFVKY	4771,179	IsaFVKY	4713,774	FsaFVKY	4615,4	YsaFVKY	2710,03
LsaFVKD	7863,834	VsaFVKD	7890,194	asaFVKD	2446,395	IsaFVKD	7567,728	FsaFVKD	10492,937	YsaFVKD	9935,447
LsaFLKE	11235,145	VsaFLKE	13731,906	asaFLKE	8188,514	IsaFLKE	12086,8	FsaFLKE	15403,398	YsaFLKE	9436,414
LsaFLKV	5862,525	VsaFLKV	7224,028	asaFLKV	3403,874	IsaFLKV	7441,791	FsaFLKV	7847,654	YsaFLKV	5566,054
LsaFLKL	7256,167	VsaFLKL	8830,896	asaFLKL	7044,44	IsaFLKL	8742,69	FsaFLKL	11283,46	YsaFLKL	6514,117
LsaFLKI	6192,481	VsaFLKI	6339,004	asaFLKI	5005,904	IsaFLKI	5584,46	FsaFLKI	10644,608	YsaFLKI	4904,32
LsaFLKa	4606,834	VsaFLKa	6648,889	asaFLKa	3853,865	IsaFLKa	7449,659	FsaFLKa	8017,048	YsaFLKa	4192,729
LsaFLKY	5168,059	VsaFLKY	4698,169	asaFLKY	3068,034	IsaFLKY	7972,57	FsaFLKY	7358,88	YsaFLKY	2969,771
LsaFLKD	9865,544	VsaFLKD	12459,443	asaFLKD	6859,536	IsaFLKD	14134,257	FsaFLKD	14890,859	YsaFLKD	8979,97
LsaFIKE	9653,533	VsaFIKE	9970,404	asaFIKE	5356,154	IsaFIKE	11342,264	FsaFIKE	14409,924	YsaFIKE	6921,341
LsaFIKV	5127,301	VsaFIKV	5591,72	asaFIKV	2490,784	IsaFIKV	6541,704	FsaFIKV	6701,9	YsaFIKV	4593,494
LsaFIKL	6430,014	VsaFIKL	7454,264	asaFIKL	4043,454	IsaFIKL	8423,19	FsaFIKL	10183,355	YsaFIKL	6100,816
LsaFIKI	5440,164	VsaFIKI	5465,879	asaFIKI	3019,831	IsaFIKI	5966,57	FsaFIKI	9055,176	YsaFIKI	5338,807
LsaFIKa	3681,794	VsaFIKa	5183,596	asaFIKa	2903,801	IsaFIKa	5886,027	FsaFIKa	8402,694	YsaFIKa	4423,536
LsaFIKY	4865,233	VsaFIKY	6194,522	asaFIKY	3537,934	IsaFIKY	6425,909	FsaFIKY	7266,72	YsaFIKY	3046,273
LsaFIKD	9603,354	VsaFIKD	9259,762	asaFIKD	3379,294	IsaFIKD	11127,584	FsaFIKD	12217,024	YsaFIKD	8341,151
LsaFaKE	8607,9686	VsaFaKE	7669,381	asaFaKE	3878,2682	IsaFaKE	9048,898	FsaFaKE	14533,251	YsaFaKE	10772,288
LsaFaKV	3767,715	VsaFaKV	2436,078	asaFaKV	1029,534	IsaFaKV	3993,618	FsaFaKV	4543,381	YsaFaKV	3077,399
LsaFaKL	4900,339	VsaFaKL	6535,325	asaFaKL	2319,923	IsaFaKL	5224,195	FsaFaKL	5337,971	YsaFaKL	3639,616
LsaFaKI	4386,981	VsaFaKI	4052,788	asaFaKI	1769,39	IsaFaKI	5589,679	FsaFaKI	3973,724	YsaFaKI	3167,14
LsaFaKa	2890,084	VsaFaKa	3433,254	asaFaKa	651,545	IsaFaKa	4869,124	FsaFaKa	4182,891	YsaFaKa	6568,281
LsaFaKY	3602,794	VsaFaKY	2545,804	asaFaKY	1422,3189	IsaFaKY	4779,545	FsaFaKY	2610,286	YsaFaKY	1789,34
LsaFaKD	6966,66	VsaFaKD	6555,4789	asaFaKD	2120,074	IsaFaKD	9977,159	FsaFaKD	8739,914	YsaFaKD	10067,649
LsaFYKE	9343,932	VsaFYKE	8676,907	asaFYKE	3802,914	IsaFYKE	11906,211	FsaFYKE	7966,511	YsaFYKE	10778,192
LsaFYKV	4029,006	VsaFYKV	3363,094	asaFYKV	2579,839	IsaFYKV	5733,996	FsaFYKV	4145,962	YsaFYKV	4340,523
LsaFYKL	5506,619	VsaFYKL	5139,944	asaFYKL	3338,735	IsaFYKL	9133,584	FsaFYKL	4722,25	YsaFYKL	4712,441
LsaFYKI	4373,904	VsaFYKI	4573,949	asaFYKI	2513,744	IsaFYKI	7131,952	FsaFYKI	4259,119	YsaFYKI	4869,896
LsaFYKa	3685,074	VsaFYKa	3566,51	asaFYKa	2438,144	IsaFYKa	5316,052	FsaFYKa	3701,374	YsaFYKa	5908,324
LsaFYKY	4515,224	VsaFYKY	4131,25	asaFYKY	2137,431	IsaFYKY	6905,724	FsaFYKY	3494,972	YsaFYKY	3614,01
LsaFYKD	7630,387	VsaFYKD	7422,566	asaFYKD	2692,32	IsaFYKD	8013,521	FsaFYKD	6148,105	YsaFYKD	7855,834
LsaFDKE	5688,572	VsaFDKE	3941,0648	asaFDKE	590,0651	IsaFDKE	10154,2922	FsaFDKE	7314,566	YsaFDKE	3692,324
LsaFDKV	7355,819	VsaFDKV	5719,382	asaFDKV	2501,228	IsaFDKV	12350,844	FsaFDKV	6473,149	YsaFDKV	3437,593
LsaFDKL	18962,144	VsaFDKL	9083,037	asaFDKL	4396,634	IsaFDKL	14259,764	FsaFDKL	7636,674	YsaFDKL	8169,974
LsaFDKI	13794,608	VsaFDKI	6603,594	asaFDKI	3445,702	IsaFDKI	9368,381	FsaFDKI	7051,084	YsaFDKI	5917,064
LsaFDKa	10130,124	VsaFDKa	6553,434	asaFDKa	3276,854	IsaFDKa	10782,484	FsaFDKa	8163,504	YsaFDKa	9528,158

LsaFDKY	8772,726	VsaFDKY	5181,483	asaFDKY	2065,873	IsaFDKY	7387,554	FsaFDKY	4489,684	YsaFDKY	4318,412
LsaFDKD	4868,4874	VsaFDKD	1771,384	asaFDKD	-77,1993	IsaFDKD	3389,6842	FsaFDKD	3303,721	YsaFDKD	2660,708
LsaYEKE	6679,6556	VsaYEKE	3517,7144	asaYEKE	511,1511	IsaYEKE	5386,138	FsaYEKE	7115,941	YsaYEKE	4750,984
LsaYEKV	6706,424	VsaYEKV	5178,671	asaYEKV	1298,454	IsaYEKV	6141,58	FsaYEKV	6000,914	YsaYEKV	4177,302
LsaYEKL	7078,108	VsaYEKL	6448,482	asaYEKL	2171,354	IsaYEKL	8252,904	FsaYEKL	11644,984	YsaYEKL	6779,613
LsaYEKI	6889,233	VsaYEKI	5698,702	asaYEKI	1949,654	IsaYEKI	6482,335	FsaYEKI	8794,383	YsaYEKI	5606,945
LsaYEKa	7383,8082	VsaYEKa	5358,454	asaYEKa	1469,248	IsaYEKa	5943,594	FsaYEKa	8618,287	YsaYEKa	5546,033
LsaYEKY	5472,214	VsaYEKY	4160,667	asaYEKY	-7,583	IsaYEKY	4808,509	FsaYEKY	6736,684	YsaYEKY	3163,205
LsaYEKD	5053,324	VsaYEKD	2339,571	asaYEKD	-111,636	IsaYEKD	4691,014	FsaYEKD	5083,789	YsaYEKD	2050,194
LsaYVKE	5141,582	VsaYVKE	5034,654	asaYVKE	2273,354	IsaYVKE	5315,25	FsaYVKE	10065,731	YsaYVKE	2861,305
LsaYVKV	4161,681	VsaYVKV	2099,988	asaYVKV	2643,139	IsaYVKV	4354,474	FsaYVKV	5307,186	YsaYVKV	2216,688
LsaYVKL	5068,495	VsaYVKL	5211,072	asaYVKL	2962,782	IsaYVKL	6248,444	FsaYVKL	8492,679	YsaYVKL	4375,681
LsaYVKI	4128,484	VsaYVKI	3600,474	asaYVKI	2438,904	IsaYVKI	6274,893	FsaYVKI	7408,53	YsaYVKI	2838,564
LsaYVKa	2871,998	VsaYVKa	4539,142	asaYVKa	1445,697	IsaYVKa	4330,367	FsaYVKa	5106,347	YsaYVKa	1871,556
LsaYVKY	2994,184	VsaYVKY	4876,517	asaYVKY	1733,819	IsaYVKY	3677,774	FsaYVKY	4476,42	YsaYVKY	2742,63
LsaYVKD	3731,748	VsaYVKD	4078,63	asaYVKD	18,918	IsaYVKD	4129,064	FsaYVKD	4167,89	YsaYVKD	1955,206
LsaYLKE	6037,98	VsaYLKE	8189,738	asaYLKE	2825,117	IsaYLKE	8699,953	FsaYLKE	8333,309	YsaYLKE	3640,695
LsaYLKV	4881,445	VsaYLKV	5919,46	asaYLKV	2612,781	IsaYLKV	6669,161	FsaYLKV	7029,68	YsaYLKV	4395,273
LsaYLKL	6705,694	VsaYLKL	7468,774	asaYLKL	3580,537	IsaYLKL	6558,84	FsaYLKL	8559,42	YsaYLKL	4737,694
LsaYLKI	5717,092	VsaYLKI	7029,991	asaYLKI	3613,194	IsaYLKI	7579,021	FsaYLKI	8246,04	YsaYLKI	3413,53
LsaYLKa	4228,016	VsaYLKa	5663,304	asaYLKa	1870,454	IsaYLKa	5261,922	FsaYLKa	3971,39	YsaYLKa	3241,447
LsaYLKY	3361,219	VsaYLKY	3789,942	asaYLKY	1527,204	IsaYLKY	4108,368	FsaYLKY	3883,165	YsaYLKY	873,244
LsaYLKD	4885,464	VsaYLKD	4440,984	asaYLKD	1760,001	IsaYLKD	7441,664	FsaYLKD	7159,714	YsaYLKD	2821,584
LsaYIKE	6585,314	VsaYIKE	12374,284	asaYIKE	2275,854	IsaYIKE	8418,485	FsaYIKE	5393,967	YsaYIKE	3687,124
LsaYIKV	5071,084	VsaYIKV	6370,824	asaYIKV	1750,087	IsaYIKV	5773,434	FsaYIKV	4763,152	YsaYIKV	2717,107
LsaYIKL	6520,624	VsaYIKL	8221,798	asaYIKL	3119,667	IsaYIKL	8901,304	FsaYIKL	6339,59	YsaYIKL	4502,104
LsaYIKI	5074,662	VsaYIKI	5075,884	asaYIKI	2580,166	IsaYIKI	6184,388	FsaYIKI	5648,527	YsaYIKI	2709,262
LsaYIKa	4022,854	VsaYIKa	5063,054	asaYIKa	1641,162	IsaYIKa	7146,052	FsaYIKa	3196,097	YsaYIKa	2792,622
LsaYIKY	3310,558	VsaYIKY	4095,475	asaYIKY	1691,844	IsaYIKY	5272,055	FsaYIKY	3477,57	YsaYIKY	2963,92
LsaYIKD	4474,843	VsaYIKD	4343,662	asaYIKD	844,787	IsaYIKD	7718,854	FsaYIKD	3089,117	YsaYIKD	2084,91
LsaYaKE	5541,161	VsaYaKE	4823,054	asaYaKE	675,62	IsaYaKE	8645,855	FsaYaKE	6003,494	YsaYaKE	4372,694
LsaYaKV	2812,502	VsaYaKV	2875,454	asaYaKV	672,885	IsaYaKV	5244,234	FsaYaKV	3659,614	YsaYaKV	1978,469
LsaYaKL	3862,894	VsaYaKL	3866,694	asaYaKL	1248,839	IsaYaKL	8223,314	FsaYaKL	4898,714	YsaYaKL	3699,764
LsaYaKI	3768,41	VsaYaKI	2764,155	asaYaKI	842,844	IsaYaKI	6446,138	FsaYaKI	4443,463	YsaYaKI	3093,856
LsaYaKa	2890,05	VsaYaKa	2768,199	asaYaKa	602,147	IsaYaKa	3719,454	FsaYaKa	4104,254	YsaYaKa	2946,709
LsaYaKY	2136,434	VsaYaKY	2917,692	asaYaKY	706,16	IsaYaKY	5497,75	FsaYaKY	3374,654	YsaYaKY	1203,744
LsaYaKD	3029,744	VsaYaKD	3347,8706	asaYaKD	111,5843	IsaYaKD	7027,7415	FsaYaKD	5083,834	YsaYaKD	2425,603
LsaYYKE	9315,015	VsaYYKE	4175,637	asaYYKE	2025,944	IsaYYKE	6122,904	FsaYYKE	6986,67	YsaYYKE	3521,84
LsaYYKV	4958,653	VsaYYKV	2975,247	asaYYKV	2096,168	IsaYYKV	4022,125	FsaYYKV	3714,82	YsaYYKV	3074,4
LsaYYKL	4716,255	VsaYYKL	4165,221	asaYYKL	3098,124	IsaYYKL	4193,165	FsaYYKL	4121,588	YsaYYKL	5564,1
LsaYYKI	4192,739	VsaYYKI	3907,001	asaYYKI	2812,008	IsaYYKI	3643,692	FsaYYKI	4204,36	YsaYYKI	4116,07
LsaYYKa	2734,274	VsaYYKa	3199,276	asaYYKa	1767,971	IsaYYKa	2952,178	FsaYYKa	3076,444	YsaYYKa	2464,874
LsaYYKY	2594,104	VsaYYKY	3264,854	asaYYKY	1631,971	IsaYYKY	2794,76	FsaYYKY	2973,73	YsaYYKY	2383,06
LsaYYKD	2821,589	VsaYYKD	2448,814	asaYYKD	226,242	IsaYYKD	2668,034	FsaYYKD	2966,105	YsaYYKD	1290,626
LsaYDKE	2481,514	VsaYDKE	500,6147	asaYDKE	-117,199	IsaYDKE	1490,212	FsaYDKE	2101,344	YsaYDKE	34,477
LsaYDKV	4452,3994	VsaYDKV	2323,822	asaYDKV	673,383	IsaYDKV	3353,713	FsaYDKV	3926,887	YsaYDKV	975,539
LsaYDKL	6198,894	VsaYDKL	3821,518	asaYDKL	1748,484	IsaYDKL	4140,548	FsaYDKL	7477,124	YsaYDKL	2300,474
LsaYDKI	5446,062	VsaYDKI	3157,489	asaYDKI	1151,879	IsaYDKI	4394,152	FsaYDKI	5816,496	YsaYDKI	2173,114
LsaYDKa	4259,081	VsaYDKa	1595,034	asaYDKa	3,678	IsaYDKa	3880,344	FsaYDKa	4230,511	YsaYDKa	890,1962
LsaYDKY	3526,384	VsaYDKY	1836,414	asaYDKY	277,9769	IsaYDKY	3709,404	FsaYDKY	3526,834	YsaYDKY	627,157
LsaYDKD	817,3261	VsaYDKD	-75,056	asaYDKD	0	IsaYDKD	954,314	FsaYDKD	843,206	YsaYDKD	-30,3926
